# Understanding In Vivo Mastication Behaviour and In Vitro Starch and Protein Digestibility of Pulsed Electric Field-Treated Black Beans after Cooking

**DOI:** 10.3390/foods10112540

**Published:** 2021-10-22

**Authors:** Marbie Alpos, Sze Ying Leong, Veronica Liesaputra, Candace E. Martin, Indrawati Oey

**Affiliations:** 1 Department of Food Science, University of Otago, Dunedin 9054, New Zealand; marbie.alpos@postgrad.otago.ac.nz (M.A.); sze.leong@otago.ac.nz (S.Y.L.); 2 Riddet Institute, Palmerston North 4442, New Zealand; 3 Department of Computer Science, University of Otago, Dunedin 9054, New Zealand; veronica.liesaputra@otago.ac.nz; 4 Department of Geology, University of Otago, Dunedin 9054, New Zealand; candace.martin@otago.ac.nz

**Keywords:** mastication, starch digestibility, protein digestibility, texture, particle size, α-amylase, black beans, calcium, pulsed electric field, legume, thermal processing, food oral processing

## Abstract

The aim of this study was to understand (i) the in vivo mastication behaviour of cooked black beans (chewing duration, texture perception, oral bolus particle size, microstructure, and salivary α-amylase) and (ii) the in vitro digestibility of starch and protein of in vivo-generated black bean oral bolus under simulated gastrointestinal condition. The beans were pre-treated using pulsed electric field (PEF) with and without calcium chloride (CaCl_2_) addition prior to cooking. The surface response model based on least square was used to optimise PEF processing condition in order to achieve the same texture properties of cooked legumes except for chewiness. In vivo mastication behaviour of the participants (*n* = 17) was characterized for the particle size of the resulting bolus, their salivary α-amylase activity, and the total chewing duration before the bolus was deemed ready for swallowing. In vitro starch and protein digestibility of the masticated bolus generated in vivo by each participant along the gastrointestinal phase were then studied. This study found two distinct groups of chewers—fast and slow chewers who masticated all black bean beans, on average, for <25 and >29 s, respectively, to achieve a bolus ready for swallowing. Longer durations of chewing resulted in boluses with small-sized particles (majorly composed of a higher number of broken-down cotyledons (2–5 mm^2^ particle size), fewer seed coats (5–13 mm^2^ particle size)), and higher activity of α-amylase. Therefore, slow chewers consistently exhibited a higher in vitro digestibility of both the starch and protein of processed black beans compared to fast chewers. Despite such distinct difference in the nutritional implication for both groups of chewers, the in vivo masticated oral bolus generated by fast chewers revealed that the processing conditions involving the PEF and addition of CaCl_2_ of black beans appeared to significantly (*p* < 0.05) enhance the in vitro digestibility of protein (by two-fold compared to untreated samples) without stimulating a considerable increase in the starch digestibility. These findings clearly demonstrated that the food structure of cooked black beans created through PEF treatment combined with masticatory action has the potential to modulate a faster hydrolysis of protein during gastrointestinal digestion, thus offering an opportunity to upgrade the quality of legume protein intake in the daily diet.

## 1. Introduction

Texture degradation of legumes due to thermal processing can be avoided by the addition of exogenous calcium to form crosslinks with demethoxylated pectins, due to activation of pectin methylesterase, to provide cell wall strengthening effect in the middle lamellae [1,2]. Recently, there is an interest to employ cell electroporation-based technology, namely pulsed electric field (PEF), to accelerate the uptake of exogenous calcium [3,4,5] and to modify the texture of plant materials [6,7]. So far, the application of PEF technology for legumes has not yet been studied, especially when this technology is used prior to hydrothermal processing. Additionally, it is not well understood how texture modification achieved using PEF technology in the presence of calcium prior to hydrothermal processing could impact the mastication behaviour of cooked legumes, and subsequently influencing the digestibility of macronutrients such as starch and protein at gastrointestinal phase.

Food digestion is a dynamic process. It starts in the mouth wherein food is broken down by the teeth through the process of chewing or mastication, which takes about 280 ms for most types of food [8]. A bolus is formed after the food is grinded to small-sized particles and lubricated by the saliva, which contains α-amylase that can hydrolyse starches. The bolus is then swallowed through the oesophagus into the stomach, where further size reduction occurs by the gastric acids, enzymatic action, and peristalsis. Food bolus is then hydrolysed in the small intestine, where most of the digestive enzymes, such as pancreatic α-amylase and proteases and brush border enzymes, are present. Bolus formation and its particle size, and the consequent digestibility of its starch and protein, can be influenced by numerous factors. This includes the properties of the food such as texture, specifically hardness, portion size, moisture and fat contents; gender, age, and the dental status of the individual chewing the food; and the mastication behaviour such as the cycle, force and duration, and saliva production [8]. Previous studies have found that food hardness greatly affects the particle size of food after mastication and softer food requires a shorter duration of mastication, resulting in larger bolus particle size [5,9]. However the effect of food chewiness is not yet much studied. Moreover, bolus particle size was reported to affect the nutrient digestibility of food such as starch, wherein larger particles, with smaller surface area, were less accessible to salivary amylase and would take a longer time to be digested at the gastrointestinal phase [10,11,12,13].

The aim of the present investigation was to understand (i) the in vivo mastication behaviour of cooked black beans (chewing duration, texture perception, oral bolus particle size, microstructure, and salivary α-amylase) and (ii) the in vitro digestibility of starch and protein of in vivo-generated black bean oral bolus under simulated gastrointestinal condition. The beans were treated using PEF processing with and without calcium chloride prior to cooking. The optimal PEF processing parameters to achieve the same texture properties of cooked legumes except for chewiness were firstly selected. One of the novelties of this study is the use of image analysis to estimate the particle size distribution of beans in the oral boluses after in vivo mastication prior to swallowing. Compared to conventional techniques, such as laser diffraction and sieving methods [14,15], image analysis is a more convenient and fast approach providing a more relevant and accurate information on the particle size of heterogenous food structure with multiple layers (e.g., seed coat), such as black beans, and irregularly shaped particles, such as masticated bolus. This technique is able to differentiate components of food when there are visible distinguishable features such as colour [13], which is highly applicable to black beans due to contrasting colours of its outer seed coat (black) and inner cotyledon (cream white). Moreover, there is very little information in the current literature on characterising the breakdown of these components during in vivo mastication of legumes.

## 2. Materials and Methods

### 2.1. Raw Material

One single batch of dried black beans (10 kg) procured from a local store was used. Upon arrival, they were sorted and those with physical damage and discoloration were excluded from the study.

### 2.2. Selection of PEF Treatment Parameters and Other Processing Variables

Different PEF processing parameters (electric field strength up to 2.3 kV/cm, specific energy up to 134 kJ/kg, pulse width of 20 µs, and frequency of 50 Hz) and calcium chloride (CaCl_2_) solution (up to 300 ppm) were systematically screened in this study. Soaked beans were placed in a PEF treatment chamber and immersed either in distilled water or in calcium chloride (CaCl_2_) solution with a bean-to-solution ratio of 1:2. The samples were PEF-treated (ELCRACK-HPV 5 PEF batch system, German Institute of Food Technologies, Quakenbrück, Germany) based on previous work [5]. The PEF chamber consisted of 2 parallel stainless-steel electrodes with an electrode distance of 80 mm. Different concentrations of calcium chloride (0 up to 300 ppm CaCl_2_ solution) resulted in differences in electrical conductivity averaged between 61 and 507 µS/cm. Pulse shape (square wave bipolar) during PEF treatment was monitored using a digital oscilloscope (UTD2042C, Uni-Trend, Dongguan City, Guangdong, China). The change in conductivity and temperature before and after PEF treatment was measured with a conductivity meter (CyberScan CON 11, Eutech Instruments, Queenstown, Singapore). The specific energy input for each PEF treatment was estimated using Equation (1).

Specific energy input (kJ/kg) = (Pulse energy of the PEF generator × Pulse number)/Total weight of black beans and CaCl_2_ solution (1)

All PEF-treated beans were then cooked at 80 °C for 60 min and their texture properties were determined. The texture profile analysis (TPA) for each cooked black bean sample type (approximately 20 g to allow at least 8–10 independent texture analyses per sample) was conducted using a texture analyser (TA-HD plus, Stable Micro Systems, Surrey, UK) based on a double compression or ‘two-bite’ test, as described by Alpos et al. [16]. The samples were compressed at 50% strain (5 beans per compression) using a 50 mm diameter cylinder probe attached in a 250 kg load cell at a test speed of 1 mm/s. The textural properties of each sample including hardness (peak force during the first compression, Newton), cohesiveness, springiness, chewiness, and resilience was determined automatically from the TPA curve using an in-house macro, developed in the Exponent software (Stable Micro Systems, Surrey, UK).

For calcium content analysis, the outer seed coat and inner cotyledon of three cooked black bean samples were individually analysed (Supplementary Materials Figure S1) due to the considerable difference in distribution of calcium in typical legumes (70% in seed coat vs. 30% in cotyledon) [17]. They were manually separated, frozen using liquid nitrogen (N_2_), freeze-dried (Labconco FreeZone freeze dryer, Kansas City, MO, USA), and then milled using a mortar and pestle to pass through a 425 µm mesh screen. The freeze-dried seed coat and cotyledon (0.1 g) were firstly digested by adding 2 mL of concentrated nitric acid (70%, *v*/*v*) in a 50 mL plastic tube. The tubes were then placed in a water bath at 95 °C for 10 min to allow complete digestion. After that, the samples were diluted to 50 mL with milli-Q water and filtered using polytetrafluoroethylene (PTFE) membrane filter (0.45 µm). Calcium concentration in the samples were determined using an inductively coupled plasma mass spectrometer (Agilent 7900 ICP-MS, Santa Clara, CA, USA). The auto tune function and helium gas mode of the instrument was utilised to minimise interferences and instrumental drift while maximising sensitivity. All samples, blanks (70% (*v*/*v*) nitric acid), and calibration standards (NIST traceable Agilent multi element standards) were introduced to the instrument in 2% (*v*/*v*) nitric acid. Scandium was used as the internal standard for calcium analysis which was added online to correct for matrix effects and instrumental drift. Calcium content measurements were performed in triplicate for each sample treatment.

A least squares model (minimizing the sum of the squares of the residuals) based on input variables (field strength, energy input, and calcium concentration) and responses (hardness, cohesiveness, springiness, chewiness, and resilience) was constructed to find the optimal settings of the input variables leading to change in chewiness, whilst preserving the other black beans texture parameters (i.e., hardness, cohesiveness, springiness, and resilience) during cooking by considering main effects, two-way interactions, and quadratic terms of the input variables. Estimates, standard error of the estimates, and *p*-value of the estimate for each model term were obtained (JMP Pro v.14, Cary, NC, USA). The “prediction profiler” feature in JMP was also employed to provide cross-sectional views of the fitted surface response and to observe how the prediction model changes across an individual input variable while holding the other inputs at fixed values.

### 2.3. Preparation of Black Bean Samples for In Vivo Mastication Study

The in vivo mastication study was conducted on three types of cooked black bean samples (i.e., shared similar texture properties except for chewiness) based on the result obtained from Section 2.2: PEF-treated in the absence of calcium chloride solution (thereafter referred as “sample A”), PEF-treated in the presence of 300 ppm CaCl_2_ solution (thereafter referred as “sample B”), and non-PEF treated black beans (thereafter referred as “sample C”). Sample A treatment involved 2000 pulses and resulted in specific energy input of 8 kJ/kg, which took 40 ms of treatment time (pulse number × pulse width). Sample B (when using 300 ppm CaCl_2_ as electrical conducting medium) underwent PEF treatment for 18 ms with 900 pulses and resulted in 13 kJ/kg specific energy input. Sample C referred to soaked bean samples that were not treated with PEF but were thermally processed in 300 ppm CaCl_2_ solution right after overnight soaking.

**Figure 1:**
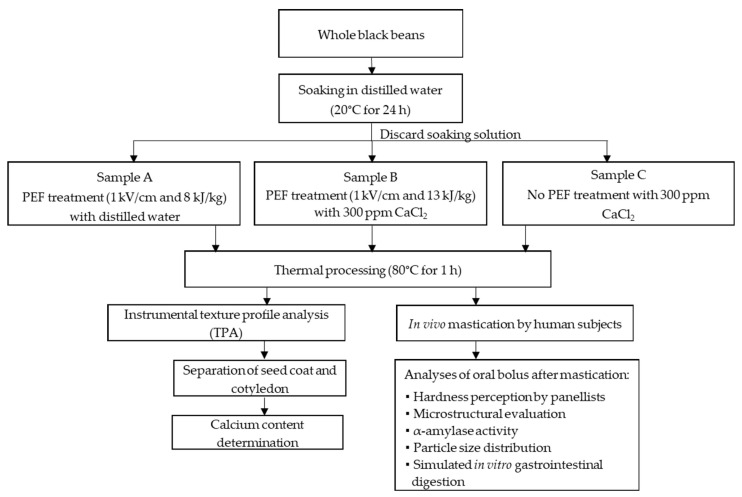
Experimental setup of this study. (PEF: Pulsed electric field).

The experimental setup of the in vivo mastication study, which comprised of sample preparation, sample collection, and subsequent analysis, is summarised in Figure 1. A day before the in vivo mastication study, black beans were soaked in distilled water (1:3 (*w*/*v*) seed-to-water ratio) for 24 h at 20 °C. The soaking water was discarded the next day. The soaked beans were divided into three lots, where the first two lots were allocated as samples A and B and proceed to PEF-treatment as described in Section 2.2. The remaining soaked beans were used as sample C. After that, all sample types were immediately thermally processed in a temperature-controlled water bath at 80 °C for 60 min. Then, samples were cooled in an ice water bath (<4 °C) for at least 20 min to remove the thermal load. The sequential PEF and thermal processing for each sample was performed in 100 g lots and then only the cooked beans (i.e., solution was removed) were pooled to obtain enough samples for the in vivo mastication study. All sample preparations were performed under food-grade conditions, ensuring adherence to food hygiene and safety protocols. Samples for each treatment were used immediately on the day for the in vivo mastication (see Section 2.4) and a small aliquot (20 g per sample) was used for texture measurement and calcium quantification (see Section 2.2) to ensure the expected texture parameters and calcium content were obtained prior to the in vivo mastication study.

### 2.4. In Vivo Mastication Study

Participants were recruited based on the following inclusion criteria: Complete dentition (without removable dentures and no dental treatments for the past 3 months), no difficulty in masticating, no pre-existing medical conditions, and no legume allergy. This study was approved by the University of Otago human ethics committee (approval reference code: 20/038) and participants were informed in detail about the objectives and methodology of the study before signing a written consent form. The results collected from 17 participants (comprising of 9 males and 8 females with a mean age of 27.2 ± 9.5 years) were analysed and reported.

The study was conducted in individual sensory booths in a well-ventilated room with fluorescent lighting. Each booth was equipped with 1 sample tray, 1 plastic spoon, a paper questionnaire, a pen, a digital timer, and a glass of drinking water (Supplementary Materials Figure S2). Every participant was provided with four separate portions of 5 g (a total of 20 g) of each sample to perform the in vivo mastication. Each participant was sequentially served (in a balanced random sample presentation order to limit first order and carry-over effects) with three separate trays for the three samples containing a total of 12 closed containers each tray (Supplementary Materials Figure S3): Four for each 5 g samples, 4 for the mouth rinsing water, and 4 for collection of the oral boluses. All containers were pre-labelled with random 3-digit codes corresponding to each sample type to prevent biased testing and it was made sure that the spitting container had the same code as the sample container.

For each sample tray, the participants were asked to randomly select one of the four sample containers and chew all the beans inside (5 g) in one mouthful until their swallowing threshold. They pressed the start button of the timer once they began chewing the sample and stopped when the sample was ready to be swallowed. The participants were not required to consume any of the sample, but to expectorate the oral boluses into the plastic spitting containers pre-labelled with the 3-digit code that matched with the sample code. The participants were then asked to rinse their mouth with the rinsing water provided (30 mL) to make sure that no food material remained in the mouth and were expectorated into the same plastic container containing the bolus. The participants were asked to repeat the same step for each of the sample containers remaining on the tray. The participants were instructed to write down, on the questionnaire, the total duration that it took for them to masticate each of the 5 g sample. After consuming all cooked beans (coming from the same process treatment) from the 4 plastic containers in the tray, the participants were asked to rate their texture perception of the sample on the questionnaire using a five-point hedonic scale [18]: very soft, soft, neither too soft nor too hard, hard, and very hard. The participants were allowed at least 30 s breaks before being presenting with the next tray of sample. The steps were repeated for the cooked beans from the second and third sample (either A, B, or C).

A total of 12 boluses were collected from each participant, 4 for each sample A, B, and C. The collected boluses, in four separate containers for each sample, were allocated for the determination of particle size distribution of bolus (see Section 2.5), α-amylase activity in the oral bolus (see Section 2.6), microstructural evaluation (see Section 2.7) and the amount of D-glucose released and L-serine in the digest when subjected to a subsequent 6 h long in vitro simulated gastrointestinal digestion (see Section 2.8). For those samples intended for the in vitro digestion, 1 mL of 1 M hydrochloric acid (HCl) was immediately added after mastication to each of the oral boluses to stop the activity of salivary amylase. All the collected boluses were frozen using liquid nitrogen (N_2_) and stored at −18 °C until analysis.

### 2.5. Particle Size Distribution of Oral Boluses Using Image Analysis

#### 2.5.1. Image Capturing and Processing of the Oral Bolus

The particle size distribution of the oral boluses across three sample types from every participant after masticating a portion of 5 g cooked black beans was determined using the image analysis technique according to the work of Bornhorst, Kostlan and Singh [13], with modifications. Each bolus was prepared by washing them on a 1 mm sieve in running tap water for 1 min to prevent the aggregation of the particles. Particles bigger than 1 mm that remained on the sieve were weighed (0.5 g) into plates (17.7 cm width) with blue-coloured background. Fifty millilitres of water were added to disperse the particles and gently stirred to prevent particles overlapping (Supplementary Materials Figure S4, left). The particles inside the entire blue plate were photographed alongside with a geometrical reference (American Board of Forensic Odontology no. 2 photomacrographic standard reference scale) for spatial calibration to convert pixels to millimetres during image processing. The camera used to capture the image was a Canon 6D Mark II (26.2 mp, Full Frame CMOS sensor, Ota City, Tokyo, Japan) with a Canon EF 50 mm f/1.7 STM lens, positioned 40 cm above the base of the blue plate. The camera was set as followed: no flash, aperture F22, ISO 3200, and shutter speed 1/200 s. Four to ten images were taken for each bolus, depending on the amounts of particles remaining on the sieve.

Captured images were analysed using the algorithm in the OpenCV software library (Open Source Computer Vision Library v.4.4.0, Palo Alto, CA, USA). The blue plate with the particles inside was first isolated using colour thresholding. Then, heuristics image analysis was performed to remove unwanted light reflection from the plate. Canny edge detection was used to identify “all” the particles in the plate and the OpenCV’s counting pixels function was used to count the total amount of pixels or the area of each identified bean particle, according to its size and shape (whether it was complex or irregular shaped). Further segmentation was conducted wherein the “white” and “black” particles, corresponding to the inner cotyledon and outer seed coat, respectively, were distinguished using *k*-means clustering method (Supplementary Materials Figure S4, right). The measured pixels of “white”, “black”, and “all” particles were converted to mm^2^ using the reference scale.

#### 2.5.2. Modelling the Particle Area Distribution of Oral Bolus

The calculated area for each particle was used to determine the cumulative particle area distribution. The cumulative area percentage was then fitted to the Rosin-Rammler model (Equation (2)) to describe the particle size distribution of the in vivo masticated black bean bolus. This model was previously used to characterise the particle size of almond and rice during oral mastication as determined by image analysis [13,19].

(2) $$C_{area}=1-\exp(-{\left(\frac{x}{x_{50}}\right)}^{b}\ln\left(2\right))$$

where *C_area_* is the cumulative area percentage of each particle from 0 to 100%, *x*_50_ is the median particle area in mm^2^, and *b* represents the dimensionless distribution breadth constant (higher *b* value corresponds to a narrower distribution spread). Nonlinear regression (function ‘nls’) in R software (v.4.0.4 2021) as commanded by R Studio (v.1.4.1103 2021, Boston, MA, USA), was used to estimate the model parameters *x*_50_ and *b* for each bolus.

### 2.6. Determination of α-Amylase Activity in Oral Boluses

The α-amylase activity in the oral boluses was determined using the Ceralpha method of the Megazyme alpha-amylase assay kit (K-CERA 06/18, Wicklow, Ireland). The procedure made use of the oligosaccharide of “non-reducing-end blocked *p*-nitrophenyl maltoheptaoside” (BPNPG7) with excess levels of thermostable α-glucosidase as substrate [20]. When α-amylase reacted with the substrate, the former cleaved a bond within the latter. Then, the excess α-glucosidase further hydrolysed the reaction product *p*-nitrophenyl maltosaccharide into glucose and free *p*-nitrophenol. The reaction was stopped by the addition of tri-sodium phosphate which changed the colour of the sample to yellow [20].

The sample was prepared by centrifugation of the oral boluses at 1000× *g* for 10 min (IEC Micromax, Thermo Electron Corp., Milford, MA, USA). The supernatant (0.05 mL) was then suitably diluted with the buffer (0.95 mL, 0.1 M sodium malate, 0.1 M sodium chloride, 4 mM calcium chloride, pH 5.4) provided in the kit. The diluted samples (0.1 mL) were transferred into bottom of 15 mL plastic tubes and pre-incubated at 40 °C for 5 min. The substrate blocked *p*-nitrophenyl maltoheptaoside (BPNPG7) was also pre-incubated (40 °C for 5 min) at the same time as the samples. After 5 min of equilibration, substrate (0.1 mL) was added directly to each sample tube, vortexed and incubated at 40 °C for another 10 min. Immediately after, 3 mL of stopping reagent (20% (*w*/*v*) tri-sodium phosphate solution, pH 11) was added and vortex mixed. The absorbance of the sample solutions and reaction blank (0.1 mL of simulated saliva juice (2 mM sodium chloride, 2 mM potassium chloride, and 25 mM sodium bicarbonate) reacted with 3 mL stopping reagent followed by addition of 0.1 mL of substrate) was measured using a spectrophotometer (Specord 250 Plus, Analytik Jena, Jena, Germany) at 400 nm against distilled water. The result was expressed as α-amylase (Ceralpha Unit, CU) in the oral boluses needed to free one micromole of *p*-nitrophenol from BPNPG7 in one minute. α-amylase measurements were performed in triplicate for each participant.

### 2.7. Microstructural Evaluation of Oral Boluses

The effect of oral mastication on the microstructure of the black beans was visualized under light microscope as described previously by Gwala et al. [21]. Briefly, boluses were lyophilised (Labconco FreeZone freeze dryer, Kansas City, MO, USA) and milled to pass through a 425 µm mesh screen. A small amount of the sample powder was dispersed in distilled water on a microscope slide, covered, and then observed under a light microscope (Ceti, Auckland, New Zealand). Micrographs were viewed at 10× magnification and captured using a camera (Medline Scientific, Oxfordshire, UK) attached to the microscope controlled by a camera control software (ToupTek ToupView, Hangzhou, Zhejiang, China).

### 2.8. Simulated In Vitro Human Gastric Intestinal Digestion Assay and Determination of Starch and Protein Digestibility

The availability of starch and protein for hydrolysis in black beans after in vivo oral mastication was determined using the harmonised static in vitro digestion method developed by Infogest [22]. The digestion solutions were freshly prepared on the day of the assay according to the work of Abduh et al. [23]. Simulated saliva juice and α-amylase solution was not added to the oral bolus sample due to prior structure breakdown and hydrolysis occurred in vivo in the mouth. Therefore, only the gastric and small intestinal digestion phases were simulated in vitro.

Twenty millilitres of simulated gastric solution (4% (*w*/*v*) porcine stomach pepsin (AppliChem A4289, 0.7 FIP-U/mg, Barcelona, Spain) in 1 mM hydrochloric acid (pH 3) containing 151 mM sodium chloride and 28 mM potassium chloride) was firstly added to the oral bolus and incubated at 37 °C (Contherm Scientific Ltd., Hutt City, Wellington, New Zealand) for 120 min with shaking (55 strokes/min, rocking motion tilt angle of 7°, DLAB, SK-R1807-S, New Territories, Hong Kong). After 2 h, pepsin was deactivated by adjusting the pH to 7 with 1 M NaOH. Then, 40 mL of simulated small intestinal solution (1% (*w*/*v*) porcine pancreas pancreatin (Sigma P1750, 4 × USP, Sigma-Aldrich, St. Louis, MO, USA) and 0.85% (*w*/*v*) g porcine bile extract (ChemCruz SC-214601, Santa Cruz Biotechnology, Dallas, TX, USA) in 0.1 M sodium bicarbonate (pH 7) was added and incubated at 37 °C for the next 240 min with shaking.

#### 2.8.1. Starch and Protein Digesta Collection and Measurement

Starch digesta (0.5 mL) were collected at 0 and 120 min of the gastric phase (after addition of simulated gastric solution) and at 0, 20, 30, 40, 60, 90, 120, 180 and 240 min of the small intestinal phase (after addition of simulated small intestinal solution). To inactivate the digestive enzymes after each sampling, the digesta were immediately heat-shocked in a boiling water bath for at least 10 min [24]. Then, to the collected digesta, 2.5 mL of 100 mL sodium acetate buffer at pH 5 were added and centrifuged at 2056× *g* (Beckman GPR Centrifuge, Brea, CA, USA) for 20 min. The amount of hydrolysed starch in the supernatant was measured using the D-glucose assay kit (Megazyme, Bray, Wicklow, Ireland) according to previous work [25,26,27]. Result expressed as amount of D-glucose in digest (mg).

In this study, different starch fractions from the in vivo-generated oral boluses of cooked black beans as digested in the small intestine were determined, which includes readily digestible starch (RDS), digested after 20 min; slowly digestible starch (SDS), digested between 20 to 120 min (slow but complete digestion); and resistant starch (RS) which passed through the small intestine undigested [28,29], using Equations (3)–(5), respectively, as follows:

(3) RDS (%)=(G20×0.9×100)/Total starch

(4) SDS (%)=((G120−G20)×0.9×100)/Total starch

(5) RS (%)=(TS−(RDS+SDS)×100)/Total starch

where G20 and G120 represent the D-glucose released after 20 and 120 min of small intestinal digestion, respectively, and 0.9 is the factor to convert the measured glucose value to polysaccharide based on the molecular mass ratio of starch to glucose (162/180) [28]. Total starch of the oral bolus was determined according to previous work [16] using the total starch assay kit (Megazyme, Bray, Wicklow, Ireland).

Protein digesta (0.5 mL) were collected at 0, 30, 60, and 120 min of the gastric phase (after addition of simulated gastric solution) and at 0, 20, 30, 40, 60, 90, 120, 180 and 240 min of the small intestinal phase (after addition of simulated small intestinal solution). They were placed in Eppendorf tubes already containing 0.5 mL of 20 (*v*/*v*%) TCA [30] and centrifuged at 13,000× *g* (IEC Micromax, Thermo Electron Corp., Milford, MA, USA) for 5 min. The *o*-phthaldialdehyde (OPA) assay, as described by Liu, Oey, Bremer, Silcock and Carne [30], was used to measure the hydrolysed protein in the oral bolus by determining the free α-amino groups of the peptide fractions, using L-serine standard curve. Results were expressed as the amount of L-serine equivalents in digest (mg).

#### 2.8.2. Kinetic Modelling of In Vitro Starch and Protein Digestibility at the Small Intestinal Phase

To estimate the extent and rate of starch hydrolysis of oral boluses, starch digestion kinetics during the small intestinal phase were modelled by a fractional conversion model (Equation (6)). The use of fractional conversion model to describe the starch digestibility behaviour of legume has been evidenced in other previous studies [11,31].

(6) $$S_{\left(t\right)}=S_{f}+(S_{0}-S_{f})\times \exp\;(k_{s}\cdot t)$$

where S_(*t*)_ is the D-glucose released at digestion time *t*, S_0_ is the amount of D-glucose at the start of small intestinal phase (0 min), S*_f_* is the D-glucose released at the end of the small intestinal digestion, and *k_s_* is the rate constant of starch digestion. The model fitting and estimation of kinetic parameters *k_s_*, S_0_, and S*_f_* were estimated using nonlinear regression function ‘nls’ in R software (v.4.0.4 2021) and R Studio (v.1.4.1103 2021, Boston, MA, USA).

Zero order kinetics model (Equation (7)) was used to estimate the extent and rate of protein hydrolysis of oral boluses during in vitro small intestinal phase.

(7) $$P_{\left(t\right)}=P_{0}+k_{p}\cdot t$$

where P_(*t*)_ is the amount of L-serine at digestion time *t*, P_0_ is the amount of L-serine at the start of small intestinal phase (0 min), and *k_p_* is the rate constant of protein digestion. The model fitting and estimation of kinetic parameter (*k_p_*) was achieved using linear regression function ‘lm’ in R software (v.4.0.4 2021) and R Studio (v.1.4.1103 2021, Boston, MA, USA).

To evaluate the goodness of fit of the kinetic models to the experimental data (starch and protein digestibility) obtained in this study, adjusted R^2^ was calculated, and residual (random distribution of error) and parity plots were assessed [21].

### 2.9. Statistical Data Analysis

Statistical analyses on the result from in vivo mastication study were performed using Statistical Package for the Social Sciences (SPSS) version 25 (IBM Corp., Armonk, NY, USA) to determine the statistical significances among sample type and participants. Data collected such as texture parameters, estimated Rosin-Rammler model parameters *x*_50_ and *b* for particle size, starch and protein digestibility, and α-amylase activity were assessed for homogeneity using Levene’s test. Then, student’s *t*-test was conducted for single comparison and analysis of variance (ANOVA) with Tukey as post hoc multiple comparison test at 0.05 level of significance. Pearson’s correlation coefficient (*r*) was also used to determine the linear relationship between the black beans texture, participants’ mastication duration, bolus particle size, salivary α-amylase activity, and starch and protein hydrolysis.

## 3. Results and Discussion

### 3.1. Selection of PEF Treatment Parameters and Other Processing Variables

From the process optimization study, pre-treating the black beans at varying levels of electric field strength and energy input in the presence of calcium did not influence their hardness, cohesiveness, springiness, and resilience after thermal processing (Supplementary Material Table S1). A similarity in the hardness result for any PEF-treated black bean samples was unexpected, since an application of PEF pre-treatment on other plant matrices has been reported to cause significant texture softening effect due the ability of PEF in disrupting the cell structure integrity [6,7,32]. It could be that the addition of calcium chloride during the PEF treatment of the black bean samples facilitated formation of crosslinks with demethoxylated pectins in the middle lamella during thermal processing, preserving their texture from thermal degradation [2].

It is interesting to observe, from the process optimization study, that the chewiness of cooked black beans was the only texture parameter affected significantly by all the input variables applied to the black beans prior to thermal processing (Supplementary Material Table S1). In other words, the chewiness of the cooked black beans can be modulated by varying the intensity of electric field strength and energy input, and concentration of calcium during PEF and thermal treatment, without compromising the other four texture parameters. The result from the response surface model also showed the potential of creating cooked black beans with similar predicted cohesiveness with increasing energy input at low field strength or high field strength at low energy input in the absence of calcium (Supplementary Material Figure S5). However, increasing the energy input further at high field strength would negatively reduce the chewiness of cooked beans without affecting the other four texture parameters. On the contrary, cooked black beans are likely to share similar cohesiveness across any levels of energy input and electric field strength in the presence of calcium during PEF (Supplementary Material Figure S6).

Considering these results, the process parameters of PEF treatments used for the in vivo mastication study were set at an electric field strength of 1 kV/cm, commonly used for plant matrix [6,33], to cause effective cell permeabilization and energy input of approximately 10 kJ/kg in the presence of 300 ppm CaCl_2_, which enabled the creation of black beans with the same hardness, cohesiveness, springiness, and resilience but not chewiness after thermal processing.

### 3.2. Texture Profile and Calcium Content of Differently Processed Black Beans Used for the In Vivo Mastication Study

From the process optimization study and response surface model, three types of black bean samples were used for the in vivo mastication study (sample A: PEF-treated and then cooked without CaCl_2_ addition, sample B: PEF-treated and then cooked in the presence of CaCl_2_, sample C: non-PEF-treated but cooked in the presence of CaCl_2_).

**Figure 2:**
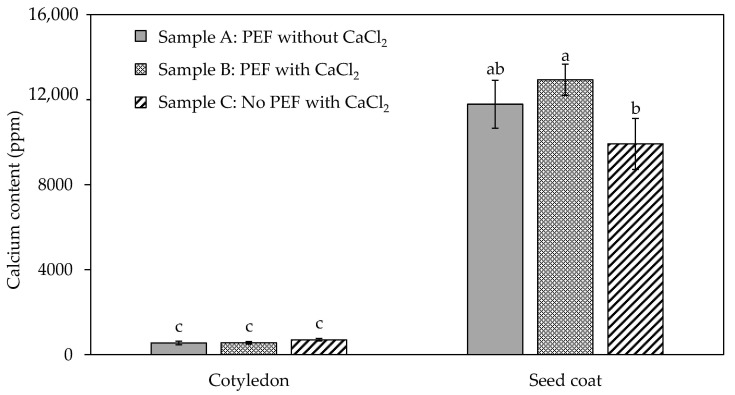
Calcium content of the cotyledon and seed coat of black bean samples A (PEF and thermally processed without CaCl_2_ addition), B (PEF and thermally processed with CaCl_2_ addition), and C (No PEF, thermally processed with CaCl_2_). Data presented as mean ± standard deviation of three independent batches of cooked black beans (*n* = 3). Values with different letters between sample type for each seed component are significantly different (*p* < 0.05).

The only texture parameter that was significantly different (*p* < 0.05) between the samples was chewiness, wherein sample B was slightly chewier than sample C (Supplementary Material Figure S7), thus requiring more energy to masticate the black beans. This can be attributed to the firming effect of calcium with improved infusion by PEF [5]. Aligning with the calcium content result of the cooked black beans (Figure 2), the addition of CaCl_2_ during PEF and thermal processing (sample B) significantly increased the calcium content in the seed coat of black beans as compared to the sample thermally processed with CaCl_2_ without PEF treatment (sample C). This indicated that PEF processing played an important role in facilitating the uptake of calcium, which is likely to occur in the testa rather than the cotyledon. Yi et al. [34] found that pectin in the cell wall of the seed coat of common beans was capable of binding to exogenous calcium ions (Ca^2+^). The increase in calcium concentration in sample B can also be explained by the ion channels present in the cell membranes of plants such as legumes which can be controlled by certain stimuli. It was known that the activation of Ca^2+^ channel, which contains calcium-selective pores, is voltage-dependent, such that application of electric pulses by PEF would open calcium channels allowing the calcium ions to pass through [35]. Another study by Zhou et al. [36] reported that the addition of CaCl_2_ (about 600 ppm) enhanced the influx and accumulation of Ca^2+^ in mung bean cells. Moreover, diffusion could have happened due to the concentration gradient. In the present study, raw black beans were soaked in water overnight and absorbed a volume twice its weight. The soaking solution was then replaced during PEF treatment with 300 ppm CaCl_2_. It can be inferred that a net movement of substance from the calcium-containing medium (higher concentration) into the beans (lower concentration) took place, as facilitated by the supposed cell electroporation effect by PEF.

Overall, results revealed that the three cooked bean samples used for the in vivo mastication study shared similarity in most of the texture properties (except for chewiness) when evaluated using the texture analyser instrument. While the application of PEF treatment in the presence of calcium enhanced the uptake of calcium in the black beans, the implications of these on the bolus formation during in vivo mastication by human participants are detailed in the following sections.

### 3.3. Characterisation of Mastication Behaviour of Participants for PEF and Calcium Pre-Treated Cooked Black Beans

#### 3.3.1. Texture Perception of Cooked Beans Rated by the Participants

The participant’s perception of hardness when masticating the three different cooked black bean samples was evaluated. Most of the participants (8–12 of them out of 17 participants) perceived all three black bean samples as “hard” on a five-point hedonic scale, followed by “neither too soft nor too hard” (Supplementary Material Figure S8). Two participants rated Sample C to be “soft” while another two participants rated both PEF-treated samples (Samples A and B) to be “very hard” (Supplementary Material Figure S8). This result suggested that instrumental measurement and consumer perception of texture could differ, because, despite the similarity in hardness of these black beans measured in the texture analyser (Supplementary Material Figure S7), participants may rate their hardness differently during consumption. Clearly, a minority of participants was rather sensitive in perceiving differences in the hardness between the cooked black bean samples due to the different pre-treatments applied. The results also suggested that it is unlikely that the chewing duration of the participants to masticate each sample to its preferable size/form prior to swallowing influences how the hardness of samples being perceived. Taking participants 10 and 12 as examples, who chewed, on average, the three types of black bean samples for 24 and 74 s, respectively—they both rated the three samples the same (“hard”), but their chewing duration varied greatly.

#### 3.3.2. Chewing Duration of Cooked Beans before Ready for Swallowing

**Figure 3:**
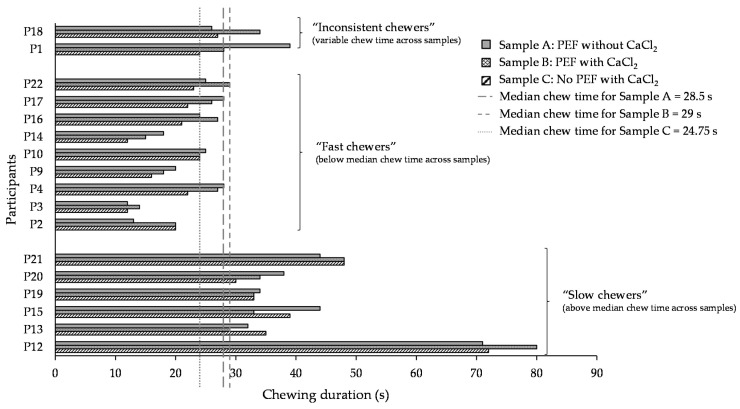
Variations in the chewing duration of participants (*n* = 17) for three different sample types (A: PEF and thermally processed without CaCl_2_ addition; B: PEF and thermally processed with CaCl_2_ addition; and C: No PEF, thermally processed with CaCl_2_). Long, short, and dotted lines correspond to the median chewing duration for each sample type. Participants with variable chewing duration across three samples are defined as “inconsistent chewers”, participants with chewing duration below the median line for all three samples are defined as “fast chewers”, and participant with chewing duration above the median line for all three samples are defined as “fast chewers”.

The total durations taken for every participant to fully masticate each sample (in one mouthful of 5 g beans), before they are about to swallow, are illustrated in Figure 3. Of all the 17 participants, the chewing duration ranged from 11.25 to 78.5 s for the three types of samples, clearly illustrating the large variation in the chewing durations between participants. The median chewing duration across the participants was 28.5, 29, and 24.75 s for samples A, B, and C, respectively (as represented by the long, short and dotted lines in Figure 3). Six participants were found to consistently take a longer chewing duration than the median duration to masticate all the samples. They were categorised as “slow chewers” in this study. On the contrary, nine participants were categorised as “fast chewers” in this study, who masticated the beans faster than the median duration. Two out of the 17 participants were considered “inconsistent chewers” due to their varying chewing pattern for each sample, specifically partly above and below the median duration. For instance, participant 18 took a shorter duration when masticating samples A and C but a longer chewing duration when masticating sample B. This was opposite for participant 1, who chewed sample A for a longer duration but samples B and C for shorter times. These results showed a great variability of chewing behaviour among individuals in this study.

Due to inter-individual variability, the average chewing duration was not significantly different (*p* > 0.05) between the three samples (Supplementary Materials Figure S9). It is recognised that humans masticate the same type of food differently [37] and individuals chew food using different oral strategies to obtain a bolus ready for swallowing. However, it cannot be ruled out that the participants misjudging the readiness of the bolus could have also contributed to the large chewing time variation. Chewing and subsequent swallowing is an act of volition; hence, it differs with individual’s preference and judgement [9].

While it is acknowledged that the chewing duration over the same sample varied greatly between participants, it was interesting to observe that sample C was chewed the fastest (averaged at 28 s) out of the three samples (averaged at 31 and 30 s for sample A and B, respectively) (Supplementary Materials Figure S9). The chewing duration positively correlated (*r* = 0.88) with the chewiness result in the texture analyser (Supplementary Material Figure S7) wherein sample C was significantly (*p* < 0.05) less chewy than sample B, which signified that the latter required more energy to masticate to be ready for swallowing, taking more time to chew. Other studies have also found a direct relationship between chewing behaviour and food texture [5,8,9,11]. Chewing time also positively correlated (*r* = 0.83) with calcium content in the seed coat (Figure 2). This indicates that, due to the lower amount of calcium found in the seed coat of sample C (Figure 2), the formation of pectin–calcium crosslinks to strengthen the cell wall was lesser than in samples A and B, making sample C easier to be chewed.

#### 3.3.3. Particle Size Distribution after In Vivo Mastication of Differently Processed Black Beans

**Figure 4:**
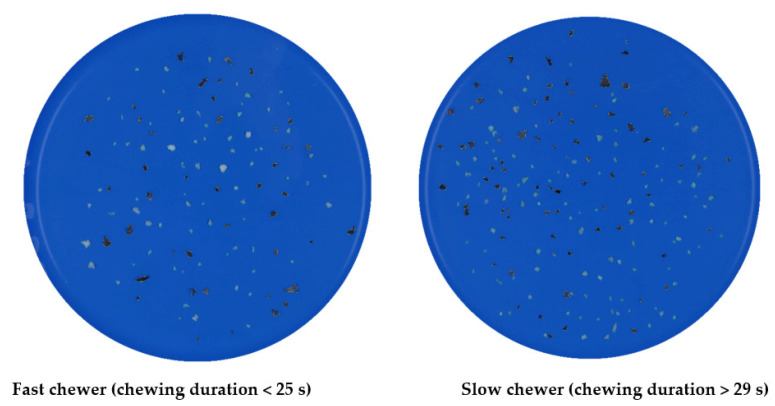
Example of processed image of black beans after mastication by a fast chewer and a slow chewer.

In this study, image analysis was found to be suitable in successfully quantifying the particle size distribution of the black beans in the oral bolus. The estimated Rosin-Rammler model parameters (*x*_50_ and *b*, Equation (2)) to describe the distribution of “all” the black bean particles, which encompassed “black” (seed coat) and “white” (cotyledon) particles (Figure 4) for each sample, are summarized in Table 1.

The median sizes of the particles (*x*_50_, indicate the 50% cumulative weight of the total food particles) for all participants ranged from 4 to 9 mm^2^ for sample A, 4 to 9 mm^2^ for sample B, and 4 to 7 mm^2^ for sample C. On average, for the 17 participants, the estimated *x*_50_ for “all” particles derived from sample A was the lowest and the highest for masticated bolus of sample C (Table 1). Likewise, the masticated bolus of sample A from all 17 participants exhibited the highest average value of estimated *b*, suggesting a narrow spread of particle distribution, compared to masticated bolus from sample C (i.e., lowest average value of estimated *b* suggesting a broad spread of particle distribution) (Table 1). However, no significant difference (*p* > 0.05) in the median particle size (*x*_50_) and distribution spread (*b*) between the samples after oral mastication was observed. The bolus particle size result was not in agreement with the texture perception by the participants, wherein sample B was majorly considered harder than the two other samples (Supplementary Material Figure S8). This shows that despite the differences in texture perception, humans are likely to masticate the samples until an acceptable bolus particle size ready to be swallowed is achieved.

In this study, one of the features utilised during the image analysis was to segregate and measure the particle size of the different components of the masticated beans, namely seed coat (“black”) and cotyledon (“white”), to better describe each of their breakdowns during mastication (Supplementary Materials Figure S4). When the particle size result was analysed based on segregating the seed coat and cotyledon of the masticated beans, it was found that a higher median particle size and a wider distribution spread (lower *b*) were observed in “black” particles than in “white” (Table 2). This can also be observed in Figure 4 (both fast and slow chewers), wherein a higher number of cotyledon (“white”) particles in bolus was evident. This was due to its larger area in the whole bean while the seed coat (“black”) particles were much bigger than cotyledon after mastication. Based on the median particle size (*x*_50_, Table 2) result and the image (Figure 4), it appeared that the participants may have difficulty breaking down the seed coat into smaller particle size to achieve a consistency that will flow smoothly during mastication as compared to the cotyledon.

With respect to the particle size differences of the oral boluses from 17 participants for three different sample types, it was found that the median particle size (*x*_50_) of “white” particles ranged from 2 to 6 mm^2^ for sample A, 2 to 5 mm^2^ for sample B, and 3 to 5 mm^2^ for sample C (Table 2). For the “black” particles, the size ranged from 5 to 11 mm^2^ for sample A, 5 to 13 mm^2^ for sample B, and 5 to 11 mm^2^ for sample C (Table 2). Although significant difference between the three samples for the *x*_50_ and *b* averaged from all 17 participants was not detected in this study, it cannot be ruled out that different processing conditions including calcium addition, PEF, and thermal processing applied to the black beans might have impact on the breakdown of particles during in vivo mastication. This is because sample C consistently showed the highest estimated *x*_50_ and the lowest estimated *b* values compared to the in vivo masticated bolus from sample A and B based on the Rosin-Rammler parameters considering “all” (Table 1), “black”, and “white” (Table 2) particles, from all 17 participants.

**Figure 5:**
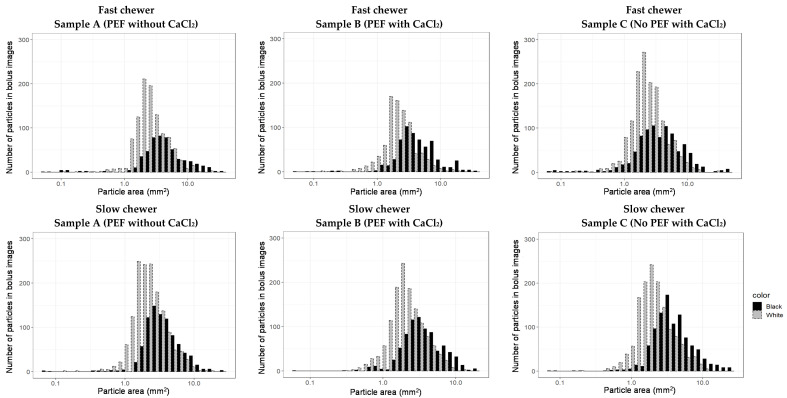
Representative examples of particle area distribution of separate components of black beans (black: seed coat, white: cotyledon) from the three samples (A: PEF and thermally processed without CaCl_2_ addition; B: PEF and thermally processed with CaCl_2_ addition; and C: No PEF, thermally processed with CaCl_2_) as masticated by a fast chewer (participant 2) and a slow chewer (participant 12).

Figure 5 visualises the particle size distribution of a slow chewer (participant 12, average of 74 s chewing duration) and a fast chewer (participant 2, average of 18 s) for each sample. The number of particles (both cotyledon and seed coat) was observed to be mostly higher in a slow chewer, who had chewed the samples longer, and thus disintegrated the particles to many smaller sizes than a fast chewer. It was also interesting to observe from Figure 5 that the histograms of particle size distribution differed for one sample to another for both representative slow and fast chewers. For example, a higher number of larger “black” particles (i.e., seed coat) of samples B and C was produced by slow chewer compared to the “white particle” of similar size from the same sample. Likewise, a similar result was exhibited by fast chewer for samples B and C. Such differences in the particle size distribution between the samples suggests that the pre-treatment applied to the black beans prior to thermal processing could play some role in influencing the way beans are disintegrating when masticated by both fast and slow chewers.

Histograms showing the distribution of the average Rosin-Rammler parameters (*x*_50_ and *b*) of “all” particles of the three black bean samples (A–C) after in vivo mastication among the participants were plotted (Supplementary Materials Figure S10) to obtain a comprehensive insight of the particle size distribution of different chewers in this study. Of the 17 participants, 53% masticated the black beans up to a median particle size of 6 mm^2^ or smaller (Supplementary Materials Figure S10). Most of them are slow chewers who chewed the three samples for longer times (>29 s), resulting in an *x*_50_ between 4.18 and 4.41 mm^2^. On the other hand, participants who chewed for <25 s (fast chewers) comminuted the beans to a larger particle size (>5 mm^2^), who resulted in oral boluses with median particle sizes of between 5.25 and 6.70 mm^2^. Moreover, the particle size distribution spread *b* was observed to be skewed to the right (Supplementary Materials Figure S10) which was attributed mostly to slow chewers. These results demonstrated that Rosin-Rammler parameters were clearly influenced by the participants’ chewing duration where *x*_50_ was found to be smaller with a higher *b* in slow chewers than in fast chewers. An increase in *b* indicates that the distribution of the particles became narrower because the participants who masticated the samples for longer duration created a bolus with uniform small particle sizes.

Although the effect of PEF processing and calcium chloride addition on the particle size distribution of in vivo masticated cooked black beans was not statistically significant, an obvious difference in the estimated Rosin-Rammler model parameters and particle size distribution between the three samples was observed (Table 1 and Table 2). Moreover, large variability in particle size between individuals was clearly exhibited in this study (Figure 5). This could be because every participant has their personal patterns of mastication and different perceptions of boluses that are deemed ready for swallowing [37]. Overall, this study revealed that median particle size (*x*_50_) and distribution spread (*b*) were found to be negatively (*r* = −0.64) and positively (*r* = 0.71) correlated, respectively, with chewing time according to Pearson’s correlation analysis. The correlation result is in agreement to the result of Olthoff et al. [38], wherein median particle size decreased as chewing strokes or duration increased.

#### 3.3.4. The Activity of α-Amylase in Oral Boluses

Apart from the food properties and chewing behaviour, bolus formation could be influenced by the amount of saliva, which contains α-amylase, an enzyme that hydrolyses starch, secreted and incorporated in the food [8]. For this reason, the activity of α-amylase in the oral bolus after mastication and expectorated by each participant was measured using the Ceralpha method of Megazyme (Section 2.6). Previous studies [39,40] have looked at the effect of food properties on saliva production wherein dry and hard foods increased the secretion of saliva to lubricate them. Values obtained in this study cannot be directly compared to other studies due to differences in assay and units used, but it would be expected that the values reported here are lower, as the spitting water was included in the bolus which could have underestimated the α-amylase activity in the saliva.

**Figure 6:**
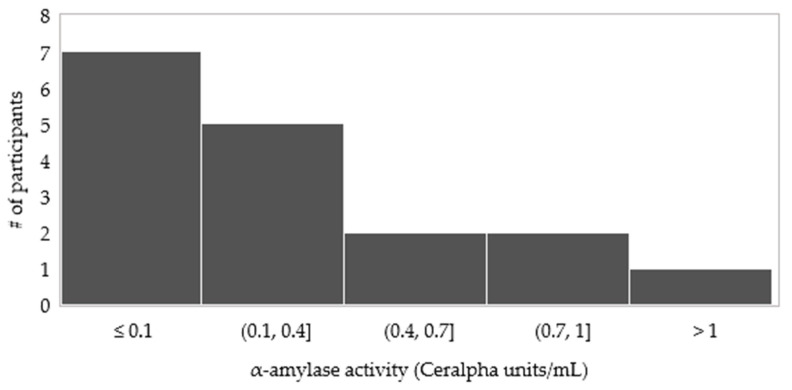
A histogram of the activity of α-amylase averaged from the oral bolus of the three samples (A: PEF and thermally processed without CaCl_2_ addition; B: PEF and thermally processed with CaCl_2_ addition; and C: No PEF, thermally processed with CaCl_2_) masticated by 17 participants.

It is not unexpected that a very wide inter-individual variation in α-amylase activity of the saliva was exhibited by the participants. For this reason, on average for 17 participants, salivary α-amylase activity in samples A, B, and C did not differ among each other (*p* > 0.05). In a way, the result suggests that cooked black beans pre-treated with PEF in the presence or absence of calcium did not affect the saliva production during human mastication. As shown in the histogram (Figure 6), the distribution of participants’ salivary α-amylase activities is skewed on the left which indicates that most of the participants have lower salivary α-amylase values. Of the 17 participants, 41% had an average α-amylase activity of ≤0.1 CU/mL. This is mostly comprised of fast chewers with α-amylase activity ranged from 0.02 to 0.01 CU/mL. Clearly, α-amylase activity in the expectorated bolus was higher in slow chewers than in fast chewers.

A positive correlation (*r* = 0.88) between chewing duration and α-amylase activity was found wherein prolonged chewing increased the amount of saliva and the activity of the enzyme. This is supported by the finding of a previous study wherein the longer the food is masticated in the mouth, the more saliva is incorporated in the bolus [9]. However, it cannot be ruled out that saliva production can vary between individuals in terms of flow rate and composition since saliva can be released in different salivary glands in the oral cavity wherein amylase-rich saliva (25% of whole saliva) is released from the parotid [8]. Overall, the results from the present study suggested that α-amylase activity increased with longer chewing duration possibly due to the increased requirement for saliva production.

#### 3.3.5. Microstructural Changes of Black Bean Oral Bolus

**Figure 7:**
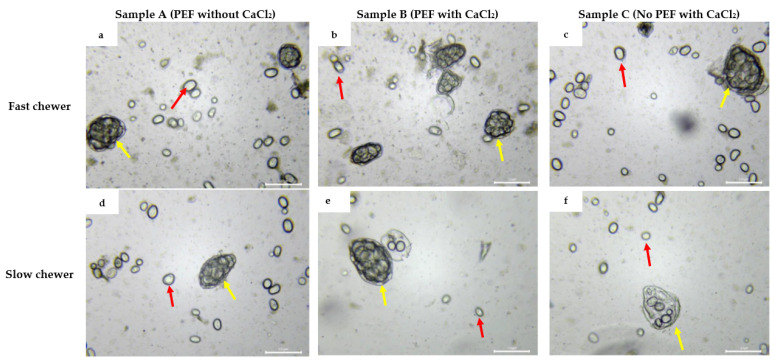
Selected light microscopic images of the starch granules from the oral bolus of three cooked black bean samples (A: PEF and thermally processed without CaCl_2_ addition; B: PEF and thermally processed with CaCl_2_ addition; and C: No PEF, thermally processed with CaCl_2_) after in vivo mastication of the fastest (**a**–**c**) and slowest (**d**–**f**) chewers. Yellow arrows show the black bean cotyledon cells and red arrows point to free starch granules. Images were viewed under 10× magnification. Scale bar = 100 µm.

Figure 7 shows the microstructural images, viewed under a light microscope, of the oral boluses of the three samples masticated by the participants within the average shortest (12 s) and longest (74 s) chewing duration. There is no remarkable difference in microstructure between samples A, B, and C as a result of varying the processing conditions. However, a more prominent change can be seen between the two chewing groups of participants. More intact cotyledon cells encapsulating the starch granules (Figure 7a–c) can be observed in the oral bolus of the fastest chewer. On the other hand, cotyledon cells that appeared to be ruptured were found in the oral bolus of the participant with the longest chewing duration with lesser amount of starch granules enclosed by the cell wall (Figure 7e,f). The presence of free starch granules was observed for both chewers (Figure 7). However, it was interesting to observe a few disrupted starch granules in the oral bolus of the slowest chewer (Supplementary Materials Figure S11). This could be a result of more shear bite forces applied to the beans with a longer duration of chewing.

The participants in this study have masticated the beans until they were ready for swallowing, hence the collected bolus will ideally be brought to the stomach for digestion. Therefore, the difference in the microstructures of the oral boluses as a result of extreme mastication duration might have an implication on the availability of starch to digestive enzymes.

### 3.4. Characterisation of the Extent of In Vitro Starch Digestibility of In Vivo Masticated Black Beans by Different Participants

#### 3.4.1. The Extent of Starch Digestibility during the In Vitro Gastric Phase

**Figure 8:**
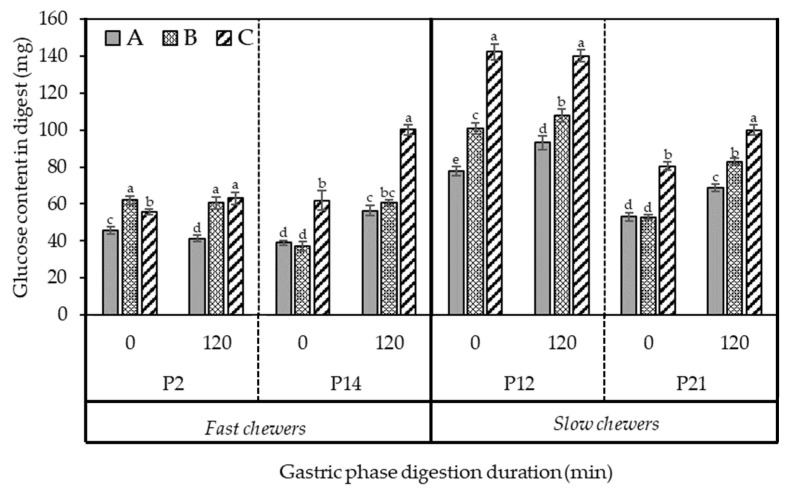
In vitro gastric starch digestion of in vivo masticated black bean samples (A: PEF and thermally processed without CaCl_2_ addition; B: PEF and thermally processed with CaCl_2_ addition; and C: No PEF, thermally processed with CaCl_2_) from selected fast and slow chewers. Data presented as mean ± standard deviation (*n* = 8 measurements). Values with different letters within each participant are significantly different (*p* < 0.05) with increasing digestion time.

Figure 8 presents the amount of D-glucose digested at the start of gastric phase (0 min, end of oral mastication) and at the completion of gastric digestion at 120 min of selected participants with different mastication behaviour, specifically fast and slow chewers.

On average, for the three samples, the amount of D-glucose released from the bolus masticated by slow chewers at the start of the gastric phase was significantly (*p* < 0.05) higher than fast chewers (Figure 8). The amount of D-glucose released at 0 min gastric phase signified the starch that have been hydrolysed in the mouth by salivary α-amylase. The increase was also consistent at the end of the gastric phase (120 min) wherein all samples masticated by slow chewers were significantly higher in digested starch. Small sized particles found in the slow chewers (Figure 4) may have provided a greater surface area for salivary fluid to lubricate and increases the susceptibility of starch granules of black beans for enzyme hydrolysis. Moreover, a higher salivary α-amylase activity found mostly in slow chewers (Figure 6) could have facilitated a greater starch hydrolysis in the small sized particles and increased the amount of available glucose even before gastric digestion has started. It is argued that the role of salivary α-amylase is considered minimal in starch hydrolysis, as compared to pancreatic α-amylase, because of its short contact time with the food in the mouth and it is readily inactivated in the gastric phase [41]. Even though salivary amylase is typically inactivated at low pH (3.3–3.8) during gastric digestion, studies have found its significant contribution in gastric digestion up to 120 min and may even remain in the small intestine [42]. Moreover, it is possible that salivary amylase can be protected from inactivation by its substrate and hydrolysis product. Earlier study of Rosenblum et al. [43] revealed that starch (0.1%), partially hydrolysed starch (5%), maltose (5%), and maltotriose (5%) provided a protection to salivary amylase from inactivation, as evidenced by the retained activity up to 90% after 120 min of gastric digestion at pH 3, which was attributed to their interaction at the active site of the enzyme.

Based on the results of individual participants, sample C appeared to be the most digestible during gastric phase compared to samples A and B due to a higher amount of D-glucose detected in the digest. However, the trend was not consistent for all the 17 participants so a direct conclusion cannot be made. When the statistical analysis of all the participants (*n* = 17) were considered, there was no significant difference (*p* > 0.05) in the amount of glucose detected in the digest between the different black bean samples A, B, and C. In other words, black beans processed using PEF, with or without calcium addition, then thermally processed, had the same starch digestibility at gastric phase as samples thermally processed alone without PEF pre-treatment. Therefore, PEF processing, which was previously determined to affect texture (Supplementary Material Figure S7) and enhance the uptake of calcium of cooked black beans (Figure 2), did not cause a considerable change in their starch digestibility at gastric phase. This indicates that PEF can potentially be used to modify the texture of legumes without posing implication on its starch hydrolysis.

#### 3.4.2. The Proportion of Different Starch Fractions (RDS, SDS and RS) Digested during the In Vitro Small Intestinal Phase

The different categories of starch in the oral boluses of three cooked black bean samples as digested in the small intestine were determined in this study. Although significant differences (*p* < 0.05) in RDS, SDS, and RS can be observed between samples (A–C) for each individual participant (Table 3 presents a few representative examples), no significant differences (*p* > 0.05) was found between samples (A–C) for all the starch fractions when the statistical analysis of all the participants (*n* = 17) was considered. This supports the result in Section 3.4.1 wherein PEF processing, even with calcium addition, does not necessarily impact the nutritional implication of legumes, specifically starch.

The mastication behaviour of the participants remained a key factor driving the proportion of starch fractions digested along the small intestinal phase. In particular, chewing duration was found to be positively correlated with RDS and SDS (*r* = 0.54 and 0.66, respectively). From Table 3, slow chewers were characterized with a significantly (*p* < 0.05) higher RDS and SDS (up to 52 and 47%, respectively) than fast chewers. On the other hand, chewing time was negatively correlated with RS (*r* = −0.61). Results showed that fast chewers who masticated the beans for shorter times, resulting in larger particle sizes, were characterized with a significantly (*p* < 0.05) higher RS (up to 67%) than slow chewers (Table 3).

These results imply that higher amounts of RDS and SDS, which are absorbed in the bloodstream, can cause an increase in blood glucose response and trigger metabolic disorder in diabetic patients [44]. On the other hand, RS which escapes the small intestine unhydrolyzed and is fermented in the large intestine by colonic microflora [45] can be beneficial in preventing type II diabetes, due to low levels of blood glucose and insulin requirement in the body [46]. Hence, it can be inferred that chewing duration and particle size can influence a foods’ nutritional functionality.

#### 3.4.3. The Kinetics of Starch Digestibility during the In Vitro Small Intestinal Phase

**Figure 9:**
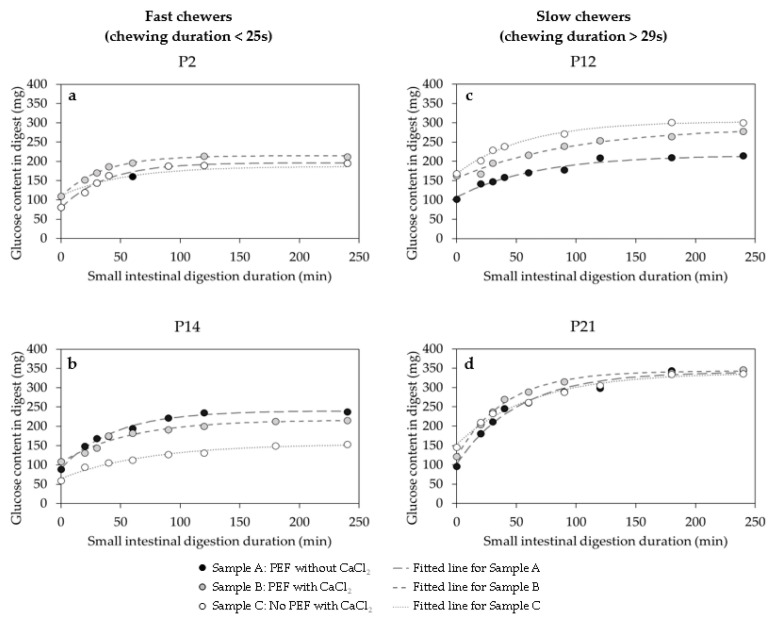
Kinetics of in vitro starch digestion (small intestinal phase) of in vivo masticated oral boluses of three cooked black bean samples (A: PEF and thermally processed without CaCl_2_ addition; B: PEF and thermally processed with CaCl_2_ addition; and C: No PEF, thermally processed with CaCl_2_) from selected fast (**a**,**b**) and slow (**c**,**d**) chewers. Experimental data are represented by circle markers and the predicted values using fractional conversion model (Equation (6)) are shown in lines.

After the gastric phase, partially hydrolysed starch enters the small intestine. Most of the starch is then hydrolysed in the small intestine due to the presence of pancreatic amylase. The digested starch kinetics (amount of D-glucose in digest plotted against small intestinal digestion time) of selected participants are shown in Figure 9. The curves exhibited an increase in D-glucose released with small intestinal digestion time followed by a plateau for all the participants, regardless of the sample type. This clearly showed that the kinetics of starch digestibility during the in vitro small intestinal phase for PEF-treated and untreated cooked black beans in the presence or absence of calcium can be described using a fractional conversion model (Equation (6)).

Three starch digestion kinetic parameters (S_0_, S*_f_*, and *k_s_*) were estimated using a fractional conversion model (Equation (6)) and the values are shown in Table 4 for selected participants categorised based on their chewing duration for each sample type. The estimated kinetic parameters describing the in vitro starch digestion were markedly varied for participants with different mastication duration. In agreement to the starch digestion kinetic curves visualized in Figure 9, slow chewers who masticated the beans for more than 29 s showed higher (up to 3-fold) estimated amounts of D-glucose released at the start (S_0_: 103–168 mg) and the end (S*_f_*: 216–343 mg) of small intestinal digestion, regardless of the sample type, than fast chewers (S_0_: 51–113 mg, S*_f_*: 134–240 mg) (Table 4). The result was expected as smaller starch granules with larger surface area from slow chewers are likely to be digested faster by amylases than larger particles with a smaller surface area from fast chewers [10]. This can be further supported by the microscopy images in Figure 7 and Supplementary Material Figure S11, wherein oral bolus of a slow chewer, predominantly, have ruptured cells and free starch granules. Free starch granules are more susceptible to amylase for digestion due to the absence of a physical barrier (cell wall) as similarly observed by Rovalino-Córdova et al. [47] in kidney beans.

It is worthy to note that the in vitro small intestinal starch kinetic curves for some participants differ for different sample type (e.g., P12 and P14, Figure 9), but there was no consistent trend showing the starch in a particular sample being digested faster or slower than other sample (Table 4). The estimated starch digestion rate constant (*k_s_*) did not vary much between the fast (1.4–3.0 × 10^−2^ min^−1^) and slow (1.0–2.5 × 10^−2^ min^−1^) chewers for the three sample types. In addition, statistical analysis taking into account all 17 participants revealed no significant difference (*p* > 0.05) between the *k_s_* of samples A–C in terms of in vitro small intestinal starch digestion. Similar with the result in the in vitro gastric phase, calcium addition combined with PEF treatment did not seem to influence starch digestion of cooked black beans at small intestinal phase. This agrees with the findings of Abduh, Leong, Agyei and Oey [23] using the same level of PEF (up to 1.1 kV/cm electric field strength) wherein in vitro gastrointestinal starch digestion of potato was not affected considerably. However, using higher intensities of PEF treatment might have a different effect. For instance, Li et al. [48] found a significant increase in in vitro digestibility of wheat, potato, and pea starches when PEF electric field strength was applied up to 8.57 kV/cm.

### 3.5. Characterisation of the Extent of In Vitro Protein Digestibility of In Vivo Masticated Black Beans by Different Participants

#### 3.5.1. The Extent of Protein Digestibility during the In Vitro Gastric Phase

**Figure 10:**
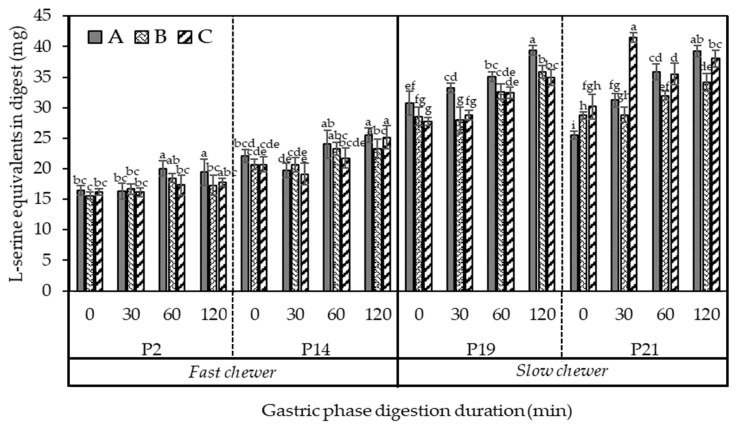
In vitro gastric protein digestion of in vivo masticated black bean samples (A: PEF and thermally processed without CaCl_2_ addition; B: PEF and thermally processed with CaCl_2_ addition; and C: No PEF, thermally processed with CaCl_2_) from selected fast and slow chewers. Data presented as mean ± standard deviation (*n* = 8 measurements). Values with different letters within each participant are significantly different (*p* < 0.05) with increasing digestion time.

Figure 10 shows the amounts of peptides or α-amino groups (calculated as L-serine equivalents) released during protein digestion in the gastric phase of oral boluses masticated by selected participants that are fast and slow chewers. Clearly, a greater increase in the protein hydrolysis was observed in slow chewers than in fast chewers along the gastric phase. At 0 min, which corresponded to the amount of available amino acids before the action of digestive enzymes in the stomach, L-serine equivalents released was up to 45% higher (on average for three samples) in slow chewers in comparison to fast chewers. This was consistent up to the end of the gastric phase (120 min), wherein slow chewers hydrolysed up to 51% more proteins than fast chewers. It should be considered that the black bean samples had been thermally processed (80 °C for 1 h) prior to in vivo mastication, which could have possibly denatured the protein and reduced antinutrients such as trypsin inhibitors owing to the free amino acids at the start of the gastric phase. Protein is further denatured in the stomach due to the acidic environment and then hydrolysed by pepsin into large polypeptides and a small number of amino acids [49]. As shown earlier in Figure 4 and Figure 7, the longer chewing time applied to the beans by slow chewers resulted in smaller particle sizes and possible ruptured cells and starch granules. This could have increased the accessibility of protein, which is bioencapsulated in the same cell compartment as starch, to proteases. A similar result in soybeans was observed wherein the degree of protein hydrolysis was increased as particle size was decreased [50]. The same study also reported a 41% increase in protein digestion when there was no physical barrier to digestive enzymes contributed by the cell wall. Nevertheless, protein digestion can still proceed in intact cells but at a slower rate. Protein digestibility was also increased in milled cowpea due to the smaller size, hence bigger surface area, for proteolytic attack [51].

Although significant differences between samples (A–C) were observed for each individual masticating the beans at each digestion time point, it was not consistent across all the participants (Figure 10). When statistical analysis considering 17 participants was conducted, the differences in protein digestibility in the gastric phase of the three samples (A–C) masticated by the participants were not significant (*p* > 0.05). Therefore, the cooked black beans pre-treated with PEF in presence or absence of calcium addition, resulted in the same degree of in vitro protein hydrolysis in the gastric phase as those processed conventionally by thermal processing. There is no known information to date on the effect of PEF on the protein digestibility of plant tissues—especially legumes—thus results cannot be validated with other studies.

#### 3.5.2. The Kinetic Behaviour of Protein Digestibility during the In Vitro Small Intestinal Phase

**Figure 11:**
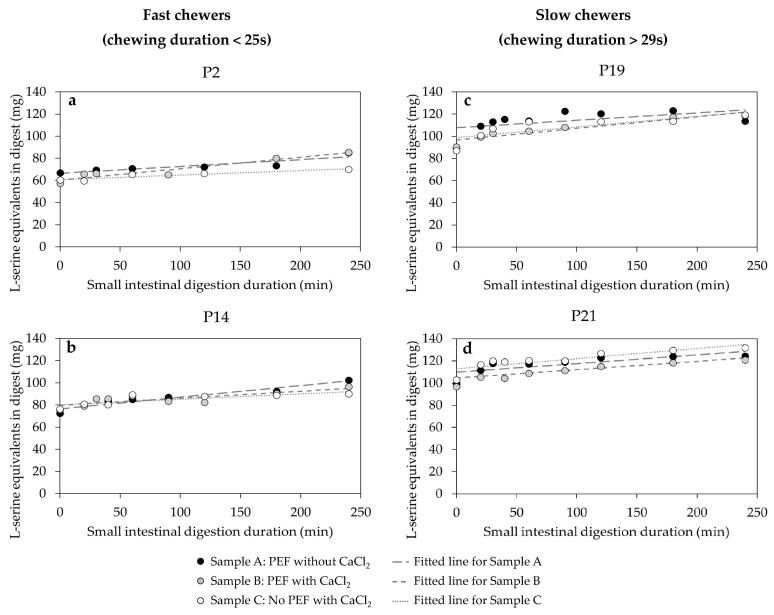
Kinetics of in vitro protein digestion (small intestinal phase) of in vivo masticated oral boluses of three cooked black bean samples (A: PEF and thermally processed without CaCl_2_ addition; B: PEF and thermally processed with CaCl_2_ addition; and C: No PEF, thermally processed with CaCl_2_) from selected fast (**a**,**b**) and slow (**c**,**d**) chewers. Experimental data are represented by circle markers and predicted values are shown in lines. All protein digestion data did not fit into a fractional conversion model and was fitted with a zero-order kinetic model (Equation (7)) instead.

Figure 11 illustrates the time-course protein hydrolysis at small intestinal phase of in vivo masticated bolus from selected fast and slow chewers fitted well with the zero-order kinetic model (Equation (7)). The amount of hydrolysed protein at the end of in vitro small intestinal phase (240 min) was up to four-fold higher than the amount digested at the end of the gastric phase (0 min), indicating that protein is mostly hydrolysed in the small intestine, by pancreatic protease and trypsin, into free amino acids and small peptide chains of two to six amino acid residues [49]. Moreover, Figure 11 shows that slow chewers have higher free amino acids or L-serine equivalents (> 100 mg) released at the start of the small intestinal phase (0 min), and even higher than at the end of the small intestinal phase (240 min) in fast chewers.

The estimated kinetic rate (*k_p_*) of protein digestion for selected participants categorised based on their chewing duration for each sample type is presented in Table 5. The rate of protein digestion was also generally higher in slow chewers (*k_p_* = 6.7–16.1 × 10^−2^ min^−1^) than in fast chewers (4.1–10.4 × 10^−2^ min^−1^). The results in this study are consistent with the starch hydrolysis result wherein nutrients in small-sized particles, as observed in the oral bolus of slow chewers due to longer chewing time (Figure 4), were more prone to enzymatic attack—this was similarly observed by Paz-Yépez et al. [52] in the protein digestibility of walnuts and peanuts. Overall, result from the current study revealed that protein digestion at small intestinal phase can be influenced by the participants’ chewing duration (*r* = 0.92) and particle size (*x*_50_) (*r* = −0.74).

Unlike the result of starch digestibility, statistical analysis revealed that protein in sample B (i.e., PEF treated with calcium then thermally processed) was digested significantly (*p* = 0.001) faster (up to 2-fold) than samples A (i.e., PEF treated without calcium then thermally processed) and C (i.e., thermally processed only in presence of calcium) in fast chewers (Table 5). In other words, PEF alone did not significantly affect the protein digestibility of black beans unless calcium was added. The cell electroporation induced by PEF of black beans potentially played a role in enhancing the digestion of protein even when calcium was added. Previous studies [30,53] found an increase in protein digestibility when egg whites and cooked beef were PEF treated (electric field strength of 0.60 kV/cm and 1.8, respectively) which was attributed to the electroporation effect and structural alteration of protein by PEF, enhancing the susceptibility of protein to digestive enzymes in the small intestine. However, calcium could also potentially contribute to the increased digestibility of protein. A recent study has found that addition of calcium (~600 ppm) enhanced the activity of phytase and acid phosphatase, which are enzymes that catalyse the hydrolysis or degradation of phytic acid [36]. Reportedly, phytic acid can lower protein digestibility by forming phytate-protein complexes with the positively charged basic amino acid of proteins [54], altering its structure and further affecting its solubility, digestibility and accessibility of protein to digestive enzymes [55,56]. However, the role of calcium in improving protein digestibility and its interaction with protein still needs further study. The results of this study are physiologically relevant because the use of PEF, a novel emerging processing, can improve protein digestion even when consumers only chew for shorter duration. This can be achieved without necessarily increasing starch digestibility which could have negative implication especially for diabetic people.

## 4. Conclusions

The current study has adopted several novel experimental and analysis approaches to understand the implication of using emerging PEF technology to process legumes on their starch and protein digestibility. The use of image analysis technique to quantify particle sizes of black beans composed of separate components (outer seed coat and inner cotyledon) has provided novel information on the breakdown of each component of untreated and PEF-treated black beans during in vivo mastication prior to swallowing. It is worthy to note that the use of emerging PEF technology on black beans was shown effective in facilitating the uptake of exogenous calcium (especially to the outer seed coat), help preventing the texture from softening during the subsequent thermal processing and able to modulate the chewiness of black beans without changing other texture parameters (i.e., hardness, cohesiveness, springiness, and resilience). Despite this, the result showed that cooked black beans with PEF pre-treatment in calcium posed no influence on the in vivo mastication behaviour among the participants that could negatively impact on the in vitro starch and protein digestibility. In fact, the observed differences in in vivo mastication behaviour between the participants, in terms of particle size, microstructure, and α-amylase activity of the resulting black beans oral bolus, was highly correlated (*r* = 0.64–0.88) with the chewing duration to masticate a mouthful of the cooked beans before it was deemed ready for swallowing. Two different groups of participants were successfully defined in this study on the basis of their chewing duration: slow (>29 s) and fast (<25 s) chewers. While the amount of protein and starch in masticated cooked black beans being digested during in vitro gastrointestinal phase was always higher in slow chewers compared to fast chewers, the combined PEF processing with CaCl_2_ addition has significantly (*p* < 0.05) increased the rate of in vitro small intestinal protein digestion of cooked black beans especially for fast chewers by two-fold. For the first time in the literature, this finding indicates an opportunity of using PEF technology on legumes to improve the digestion of protein for a certain consumer group, such as fast chewers without triggering an increase in the starch digestibility. Clearly, PEF treatment application on black beans is shown promising to hasten the release and hydrolysis of protein in the gastrointestinal tract, but it is of future interest to investigate whether the hydrolysed proteins become more bioaccessible and then available for absorption in the blood stream to carry the relevant metabolic function in the body. Forthcoming studies should also address whether PEF treatment can be applied on other legume (types and physical structures, e.g., without outer seed coat) to facilitate release of nutritious high-quality plant proteins.

## Figures and Tables

**Table 1:** Estimated Rosin-Rammler parameters (Equation (2)) of “all” black bean particles from samples A (PEF and thermally processed without CaCl_2_ addition), B (PEF and thermally processed with CaCl_2_ addition), and C (No PEF, thermally processed with CaCl_2_) after in vivo human mastication (average of 17 participants).

Sample	Oral Bolus ^†^	
	*x*_50_ (mm^2^)	*b*
A	5.0 ± 1.3 ^a^	1.63 ± 0.19 ^a^
B	5.2 ± 1.2 ^a^	1.60 ± 0.18 ^a^
C	5.4 ± 0.9 ^a^	1.53 ± 0.14 ^a^

^†^ Data are presented as the average ± standard deviation of the estimated value from 17 participants (*n* = 17) reporting according to the significant figures. Values with the same lowercase letters in superscript for the three samples within each parameter (per column) are not significantly different (*p* > 0.05).

**Table 2:** Estimated Rosin-Rammler parameters (Equation (2)) of the particles of black bean cotyledon (“white”) and seed coat (“black”) from samples A (PEF and thermally processed without CaCl_2_ addition), B (PEF and thermally processed with CaCl_2_ addition), and C (No PEF, thermally processed with CaCl_2_) after in vivo human mastication (average of 17 participants).

Sample	Cotyledon (“White”)		Seed Coat (“Black”)	
	*x*_50_ (mm^2^)	*b*	*x*_50_ (mm^2^)	*b*
A	3.5 ± 0.9 ^a^	1.9 ± 0.3 ^a^	6.7 ± 1.8 ^a^	1.7 ± 0.2 ^a^
B	3.5 ± 0.8 ^a^	1.9 ± 0.3 ^a^	7.1 ± 2.0 ^a^	1.7 ± 0.3 ^a^
C	3.7 ± 0.6 ^a^	1.8 ± 0.3 ^a^	7.6 ± 1.4 ^a^	1.58 ± 0.16 ^a^

Data are presented as the average ± standard deviation of the estimated value from 17 participants (*n* = 17) reporting according to the significant figures. Values with the same lowercase letters in superscript for the three samples within each parameter (per column) are not significantly different (*p* > 0.05).

**Table 3:** Starch fractions of in vivo masticated boluses of the black bean samples (A: PEF and thermally processed without CaCl_2_ addition; B: PEF and thermally processed with CaCl_2_ addition; and C: No PEF, thermally processed with CaCl_2_) from selected slow and fast chewers after in vitro small intestinal digestion.

Participants	RDS (%)			SDS (%)			RS (%)		
	Sample A	Sample B	Sample C	Sample A	Sample B	Sample C	Sample A	Sample B	Sample C
**Fast chewers (chewed < 25 s)**									
P2	32.8 ± 0.7 ^d^_C_	42.91 ± 1.70 ^d^_A_	38.6 ± 0.9 ^d^_B_	19.5 ± 2.4 ^c^_A_	17.6 ± 1.8 ^b^_A_	18.6 ± 7.0 ^cd^_A_	47.8 ± 1.9 ^b^_A_	39.52 ± 1.80 ^c^_B_	42.9 ± 6.8 ^c^_AB_
P14	37.7 ± 1.9 ^c^_A_	37.0 ± 2.0 ^e^_A_	27.7 ± 1.1 ^f^_B_	21.8 ± 0.9 ^c^_A_	19.3 ± 0.9 ^b^_B_	10.9 ± 1.7 ^e^_C_	40.5 ± 1.4 ^c^_C_	43.71 ± 2.50 ^b^_B_	61.32 ± 1.10 ^a^_A_
P16	20.81 ± 0.70 ^e^_C_	29.6 ± 0.6 ^f^_B_	34.0 ± 1.3 ^e^_A_	13.6 ± 1.3 ^d^_B_	18.6 ± 1.8 ^b^_A_	13.2 ± 1.7 ^de^_B_	65.6 ± 1.1 ^a^_A_	51.8 ± 1.7 ^a^_B_	52.8 ± 1.2 ^b^_B_
**Slow chewers (chewed > 29 s)**									
P12	39.0 ± 1.5 ^c^_C_	47.8 ± 1.3 ^c^_B_	57.4 ± 2.3 ^b^_A_	26.2 ± 3.5 ^b^_A_	24.5 ± 0.9 ^a^_A_	19.6 ± 1.9 ^bc^_B_	33.4 ± 3.3 ^d^_A_	27.7 ± 0.9 ^d^_B_	23.0 ± 2.5 ^d^_C_
P19	56.4 ± 1.0 ^a^_B_	69.1 ± 1.1 ^a^_A_	51.6 ± 0.9 ^c^_C_	26.4 ± 1.4 ^b^_A_	19.1 ± 2.2 ^b^_B_	24.3 ± 2.3 ^ab^_A_	17.2 ± 1.0 ^f^_B_	13.6 ± 1.8 ^f^_C_	24.2 ± 1.7 ^d^_A_
P21	46.2 ± 1.4 ^b^_C_	54.9 ± 2.2 ^b^_B_	61.8 ± 1.5 ^a^_A_	29.9 ± 3.1 ^a^_A_	27.4 ± 3.2 ^a^_A_	28.3 ± 3.9 ^a^_A_	24.0 ± 1.9 ^e^_A_	17.7 ± 2.1 ^e^_B_	11.7 ± 1.5 ^e^_C_

Data presented as mean ± standard deviation (*n* = 8 measurements) reporting according to the significant figures. Values with different lowercase letters in superscript are significantly different (*p* < 0.05) between participants for each starch fraction (RDS: readily digestible starch, SDS: slowly digestible starch, and RS: resistant starch). Values with different uppercase letters in subscript are significantly different (*p* < 0.05) between black bean samples (A, B, and C) for each participant within the same starch fraction.

**Table 4:** Kinetic parameters of in vitro small intestinal starch digestion of in vivo masticated oral boluses of the black bean samples (A: PEF and thermally processed without CaCl_2_ addition; B: PEF and thermally processed with CaCl_2_ addition; and C: No PEF, thermally processed with CaCl_2_) from selected fast and slow chewers as estimated using fractional conversion model (Equation (6)).

Participants	S_0_ (mg)			S*_f_* (mg)			*k_s_* (×10^−2^ min^−1^)		
	Sample A	Sample B	Sample C	Sample A	Sample B	Sample C	Sample A	Sample B	Sample C
**Fast chewers (chewed < 25 s)**									
P2	78.4 ± 5.6	108.5 ± 3.3	110.6 ± 7.2	196.4 ± 4.4	214.6 ± 2.7	186.9 ± 6.7	2.7 ± 0.4	3.0 ± 0.2	1.9 ± 0.6
P14	90.5 ± 5.2	105.9 ± 6.6	64.4 ± 4.9	240.0 ± 4.7	216.0 ± 6.1	154.6 ± 6.1	2.3 ± 0.2	1.8 ± 0.3	1.4 ± 0.3
P16	50.6 ± 4.4	112.5 ± 4.2	103.4 ± 5.0	133.8 ± 4.5	209.5 ± 4.8	200.9 ± 4.6	1.6 ± 0.3	1.4 ± 0.2	1.8 ± 0.3
Average	73.18 ± 5.06 ^a^	109.0 ± 4.7 ^a^	92.8 ± 5.7 ^a^	190.1 ± 4.6 ^a^	213.4 ± 4.5 ^a^	180.8 ± 5.8 ^a^	2.2 ± 0.3 ^a^	2.1 ± 0.3 ^a^	1.7 ± 0.4 ^a^
**Slow chewers (chewed > 29 s)**									
P12	107.3 ± 5.4	156.2 ± 6.1	168.0 ± 4.7	216.0 ± 6.2	289.2 ± 12.3	303.8 ± 4.5	1.5 ± 0.2	1.0 ± 0.2	1.8 ± 0.2
P19	152.3 ± 7.8	145.1 ± 11.9	128.6 ± 11.8	324.8 ± 5.7	306.40 ± 9.80	306.5 ± 9.3	2.3 ± 0.3	2.1 ± 0.4	2.1 ± 0.4
P21	102.6 ± 11.6	121.6 ± 5.2	152.9 ± 7.1	340.7 ± 9.9	343.0 ± 3.9	338.8 ± 7.7	1.9 ± 0.3	2.5 ± 0.1	1.6 ± 0.2
Average	120.7 ± 8.3 ^a^	141.0 ± 7.7 ^a^	149.8 ± 6.3 ^a^	293.8 ± 7.3 ^a^	312.9 ± 6.5 ^a^	316.4 ± 7.2 ^a^	1.9 ± 0.3 ^a^	1.9 ± 0.3 ^a^	1.8 ± 0.3 ^a^

Data presented as estimated value ± standard error of the model (Equation (6)) reporting according to the significant figures. Average values with different lowercase letters in superscript are significantly different (*p* < 0.05) between samples (A–C) for each group of chewers within the same kinetic parameter. S_0_: D-glucose released at the start of small intestinal digestion, S*_f_*: D-glucose released at the end of small intestinal digestion, and *k_s_*: estimated starch digestion rate constant. P2, P14, P16, P12, P19, and P21 corresponds to participants’ numbers.

**Table 5:** Estimated rate of in vitro small intestinal protein digestion *k_p_* (×10^−2^ min^−1^) of in vivo masticated oral boluses of the black bean samples (A: PEF and thermally processed without CaCl_2_ addition; B: PEF and thermally processed with CaCl_2_ addition; and C: No PEF, thermally processed with CaCl_2_) from selected fast and slow chewers.

Participants	Sample A	Sample B	Sample C
**Fast chewers (chewed < 25 s)**			
P2	6.2 ± 1.5	10.4 ± 1.5	4.1 ± 0.9
P4	5.5 ± 1.6	9.2 ± 1.0	4.3 ± 1.9
P16	4.7 ± 1.5	8.4 ± 1.3	5.4 ± 1.8
Average	5.5 ± 1.5 ^b^	9.3 ± 1.3 ^a^	4.6 ± 1.5 ^b^
**Slow chewers (chewed > 29 s)**			
P12	10.2 ± 1.5	16.1 ± 1.2	11.5 ± 2.7
P19	6.7 ± 4.0	10.5 ± 1.7	9.6 ± 3.3
P21	7.7 ± 2.5	7.5 ± 2.6	9.0 ± 2.1
Average	8.2 ± 2.7 ^a^	11.3 ± 1.8 ^a^	10.0 ± 2.7 ^a^

Data presented as estimated value ± standard error of the model (Equation (7)) reporting according to the significant figures. Average values with different lowercase letters in superscript are significantly different (*p* < 0.05) between samples (A–C) for each group of chewers. P2, P4, P16, P12, P19, and P21 corresponds to participants’ numbers.

## Supplementary Materials

The following are available online at https://www.mdpi.com/article/10.3390/foods10112540/s1, Figure S1: Black beans outer seed coat (blue arrow) and inner cotyledon (orange arrow) separated for calcium content analysis, Figure S2: Sensory booth set up for each participant during the in vivo mastication study, Figure S3: Preparation of separate trays for the three samples presented to the participants containing the sample containers (5 g sample per container, blue arrow), mouth rinsing water containers (30 mL per container, black arrow), and spitting containers (green arrow), Figure S4: Sample image processing of black beans oral bolus. Left: black beans dispersed in the plate and positioned for picture taking with the sample label and reference scale. Right: after image processing showing the segmentation of the “white” (marked green) and “black” (marked yellow) particles, Figure S5: Traces of prediction profile (from JMP Pro v.14) showing the predicted texture parameters of cooked black beans as either PEF electric field strength or energy input is changed, while the calcium concentration is held constant at 0 ppm, Figure S6: Traces of prediction profile (from JMP Pro v.14) showing the predicted texture parameters of cooked black beans as either PEF electric field strength or energy input is changed while the calcium concentration is held constant at 300 ppm, Figure S7: Texture parameters of the selected three black bean samples (A: PEF and thermally processed without CaCl_2_ addition, B: PEF and thermally processed with CaCl_2_ addition, and C: No PEF, thermally processed with CaCl_2_) used for in vivo oral mastication study. Data presented as mean ± standard deviation of forty-eight independent measurements (*n* = 48). Values with different lowercase letters between sample type for each texture parameter are significantly different (*p* < 0.05), Figure S8: Participants (*n* = 17) perception of the hardness of three black bean samples (A: PEF and thermally processed without CaCl_2_ addition; B: PEF and thermally processed with CaCl_2_ addition; and C: No PEF, thermally processed with CaCl_2_) rated on a five-point hedonic scale, Figure S9: Boxplot of the chewing duration of the three samples (A: PEF and thermally processed without CaCl_2_ addition; B: PEF and thermally processed with CaCl_2_ addition; and C: No PEF, thermally processed with CaCl_2_) by 17 participants. Upper and lower lines of the box are the upper and lower quartiles, the lines inside the box represent the median values, and the lines extending outside of the box are the minimum and maximum values. No significant difference (*p* > 0.05) was observed between samples, Figure S10: Histograms showing the distribution of the average Rosin-Rammler parameters (*x*_50_ and *b*) of “all” particles of the three black bean samples (A: PEF and thermally processed without CaCl_2_ addition; B: PEF and thermally processed with CaCl_2_ addition; and C: No PEF, thermally processed with CaCl_2_) after in vivo mastication (*n* = 17), Figure S11: Light microscopic image showing broken free starch granules from the oral bolus of a slow chewer. Images were viewed under 50× magnification. Scale bar = 100 μm, Table S1: *p*-value for the estimates of the model parameters examining the response surface effect of input variables (PEF electric field strength, PEF energy input, and calcium concentration during PEF and thermal processing) against texture parameters of cooked black beans (hardness, cohesiveness, springiness, chewiness, and resilience).
