# Supplementary material for: Understanding In Vivo Mastication Behaviour and In Vitro Starch and Protein Digestibility of Pulsed Electric Field-Treated Black Beans after Cooking
Source: Foods. 2021 Oct 22;10(11):2540. doi: 10.3390/foods10112540 (PMC8622574; doi:10.3390/foods10112540)
Supplement: Supplementary file 1 [file foods-10-02540-s001.zip › foods-1382455-supplementary.pdf]

foods MDPI

Supplementary Materials

Understanding *In Vivo* Mastication Behaviour and *In Vitro* Starch and Protein Digestibility of Pulsed Electric Field treated Black Beans after Cooking

Marbie Alpos, Sze Ying Leong, Veronica Liesaputra, Candace E. Martin and Indrawati Oey*
Correspondence: Department of Food Science, University of Otago, Dunedin 9054, New Zealand
indrawati.oey@otago.ac.nz; Tel.: +64-3-479-8735

Figure S1. Black beans outer seed coat (blue arrow) and inner cotyledon (orange arrow) separated for calcium content analysis.

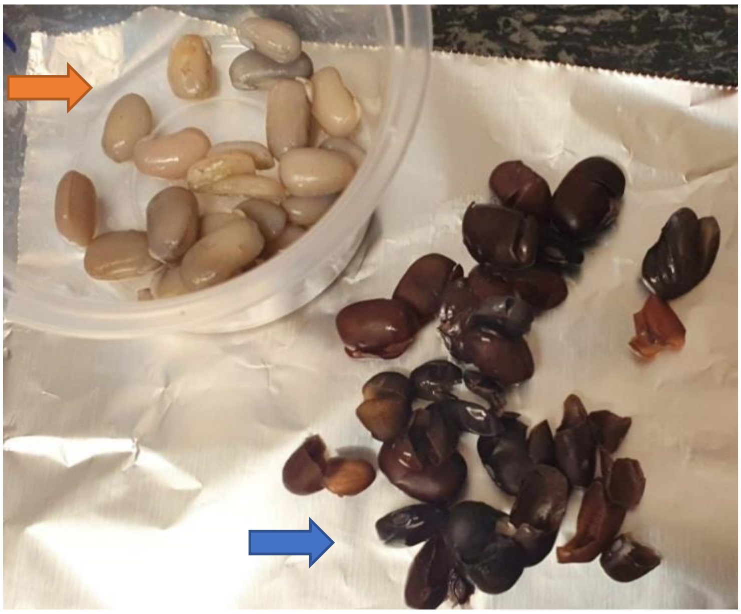

Figure S2. Sensory booth set up for each participant during the in vivo mastication study.

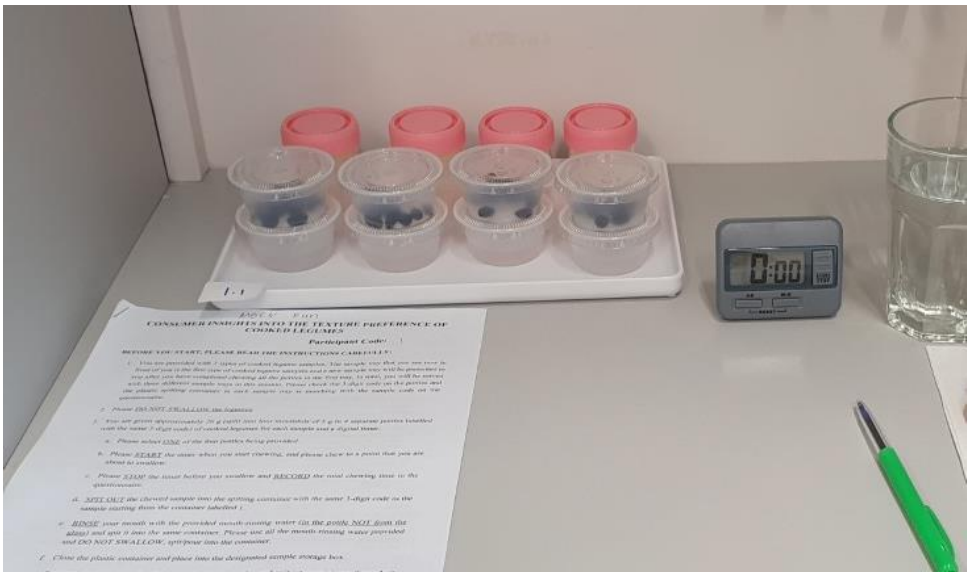

Figure S3. Preparation of separate trays for the three samples presented to the participants containing the sample containers (5 g sample per container, blue arrow), mouth rinsing water containers (30 mL per container, black arrow), and spitting containers (green arrow).

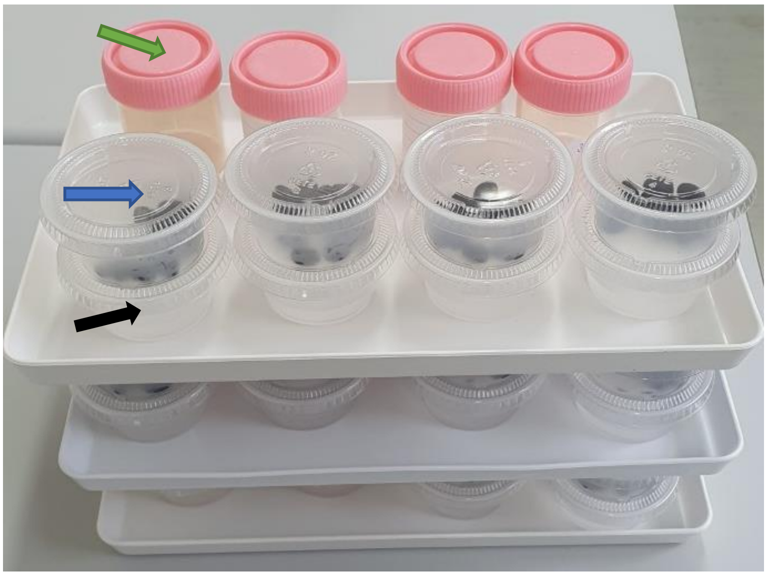

Figure S4. Sample image processing of black beans oral bolus. Left: black beans dispersed in the plate and positioned for picture taking with the sample label and reference scale. Right: after image processing showing the segmentation of the “white” (marked green) and “black” (marked yellow) particles.

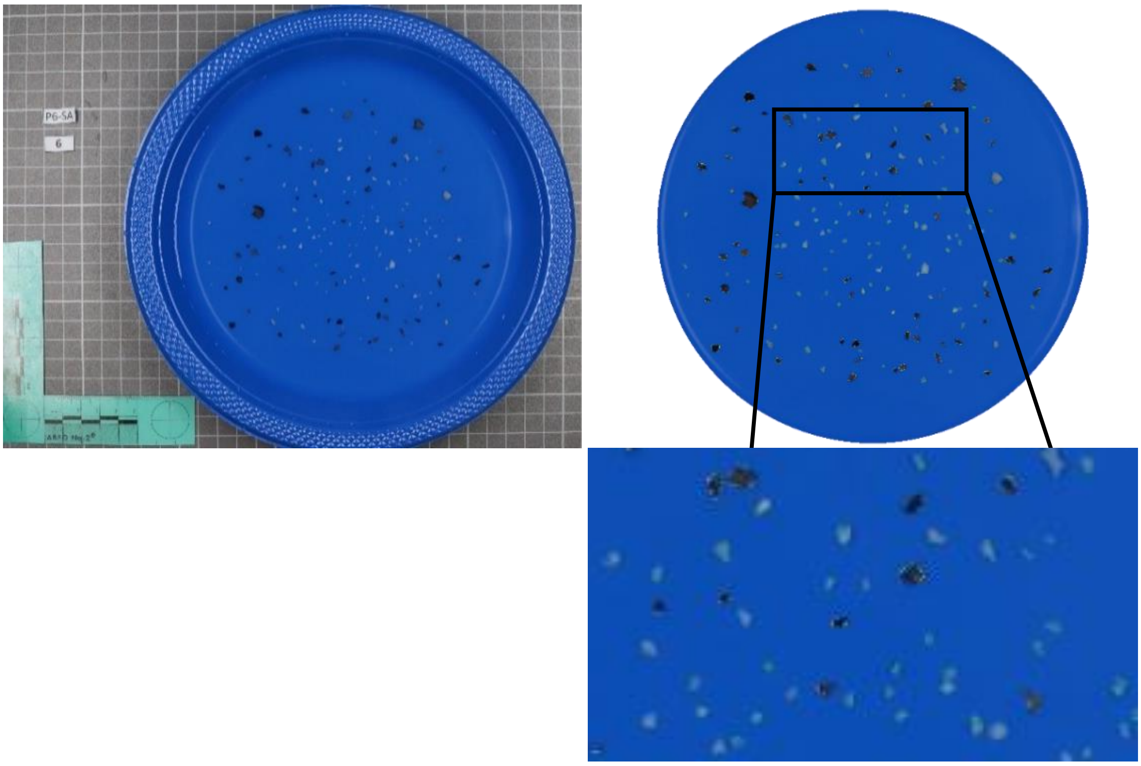

Figure S5. Traces of prediction profile (from JMP Pro v.14) showing the predicted texture parameters of cooked black beans as either PEF electric field strength or energy input is changed while the calcium concentration is held constant at 0 ppm.

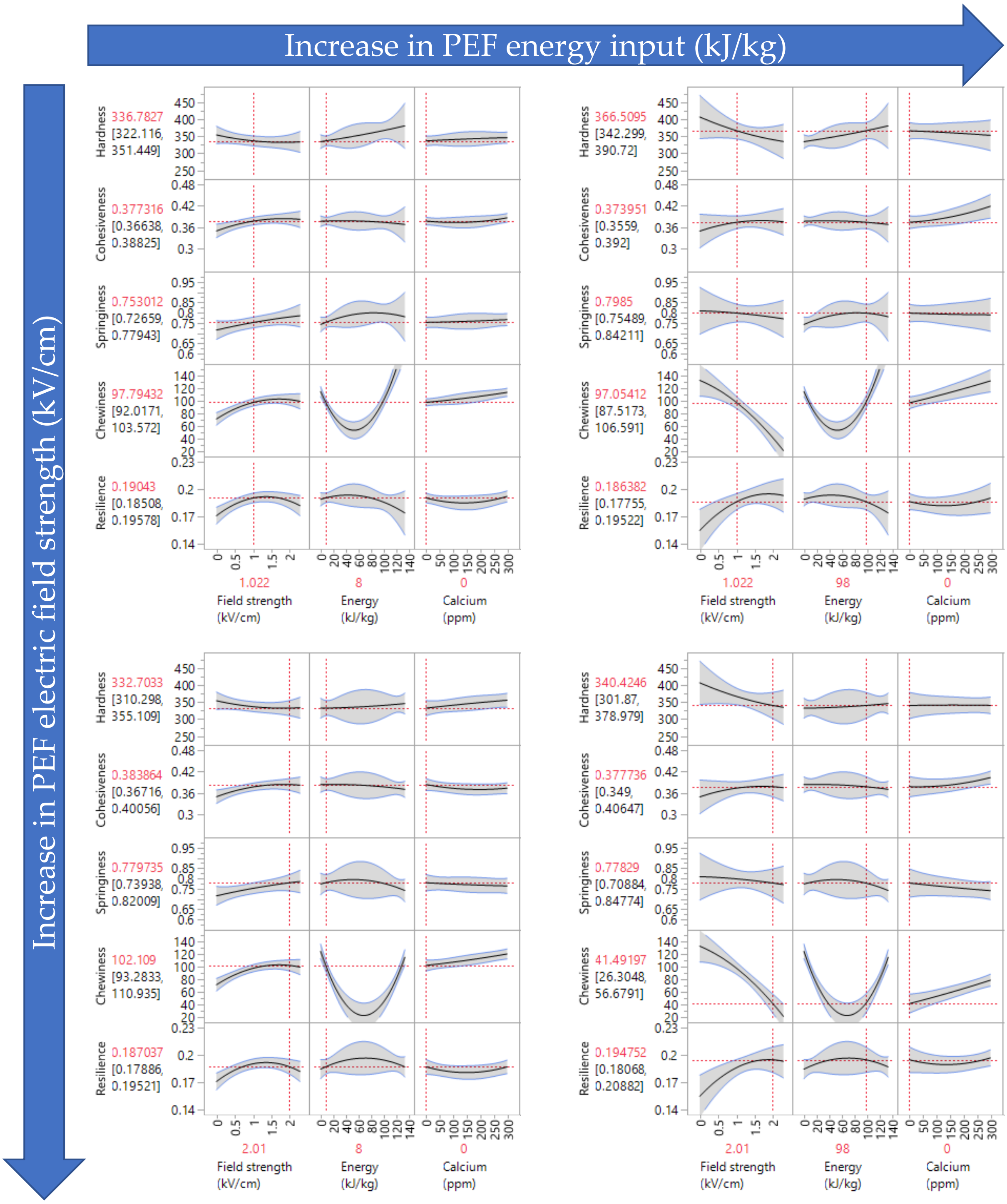

Figure S6. Traces of prediction profile (from JMP Pro v.14) showing the predicted texture parameters of cooked black beans as either PEF electric field strength or energy input is changed while the calcium concentration is held constant at 300 ppm.

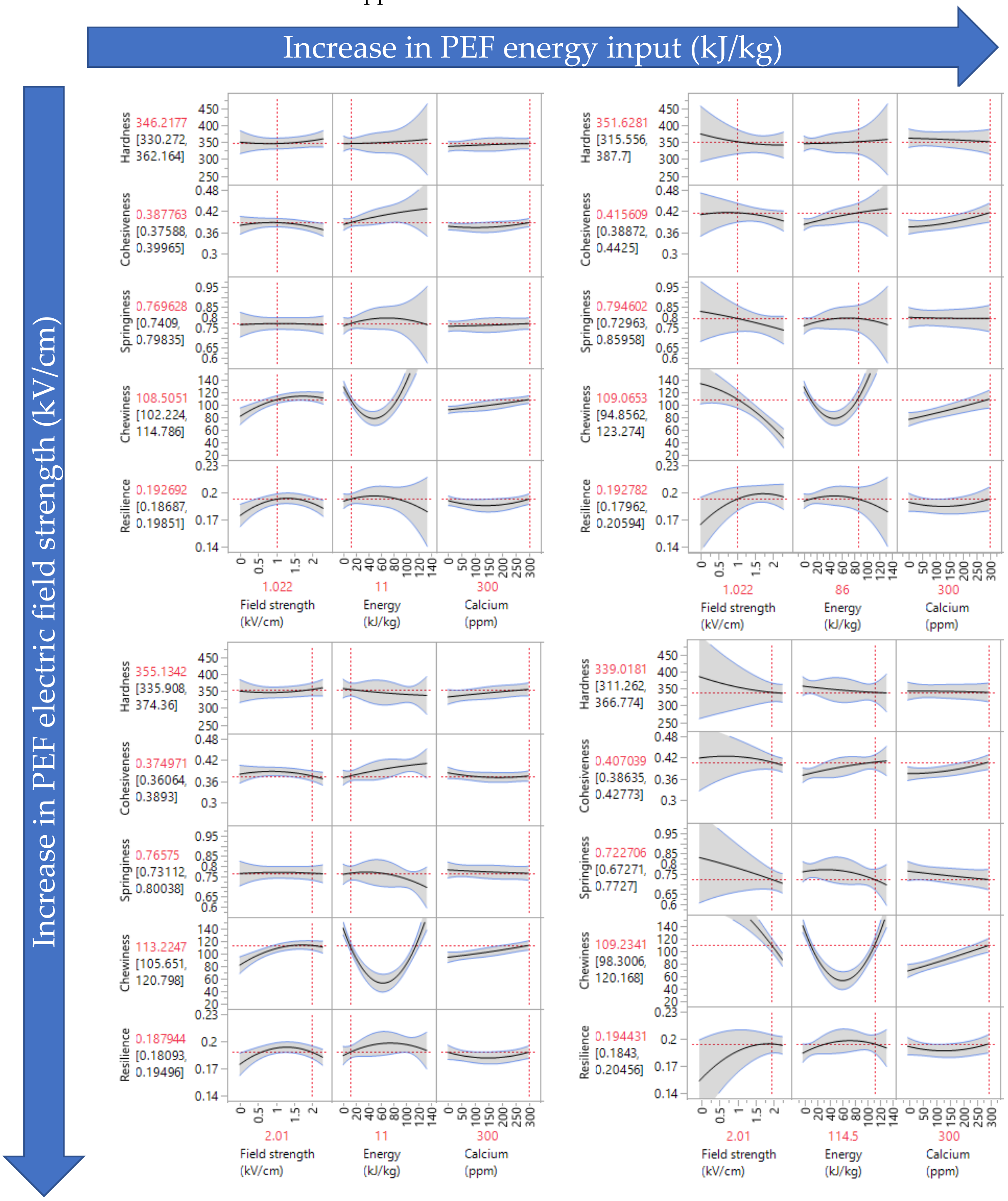

Figure S7. Texture parameters of the selected three black bean samples (A: PEF and thermally processed without $CaCl_2$ addition, B: PEF and thermally processed with $CaCl_2$ addition, and C: No PEF, thermally processed with $CaCl_2$) used for in vivo oral mastication study. Data presented as mean ± standard deviation of forty-eight independent measurements (n=48). Values with different lowercase letters between sample type for each texture parameter are significantly different ($p < 0.05$).

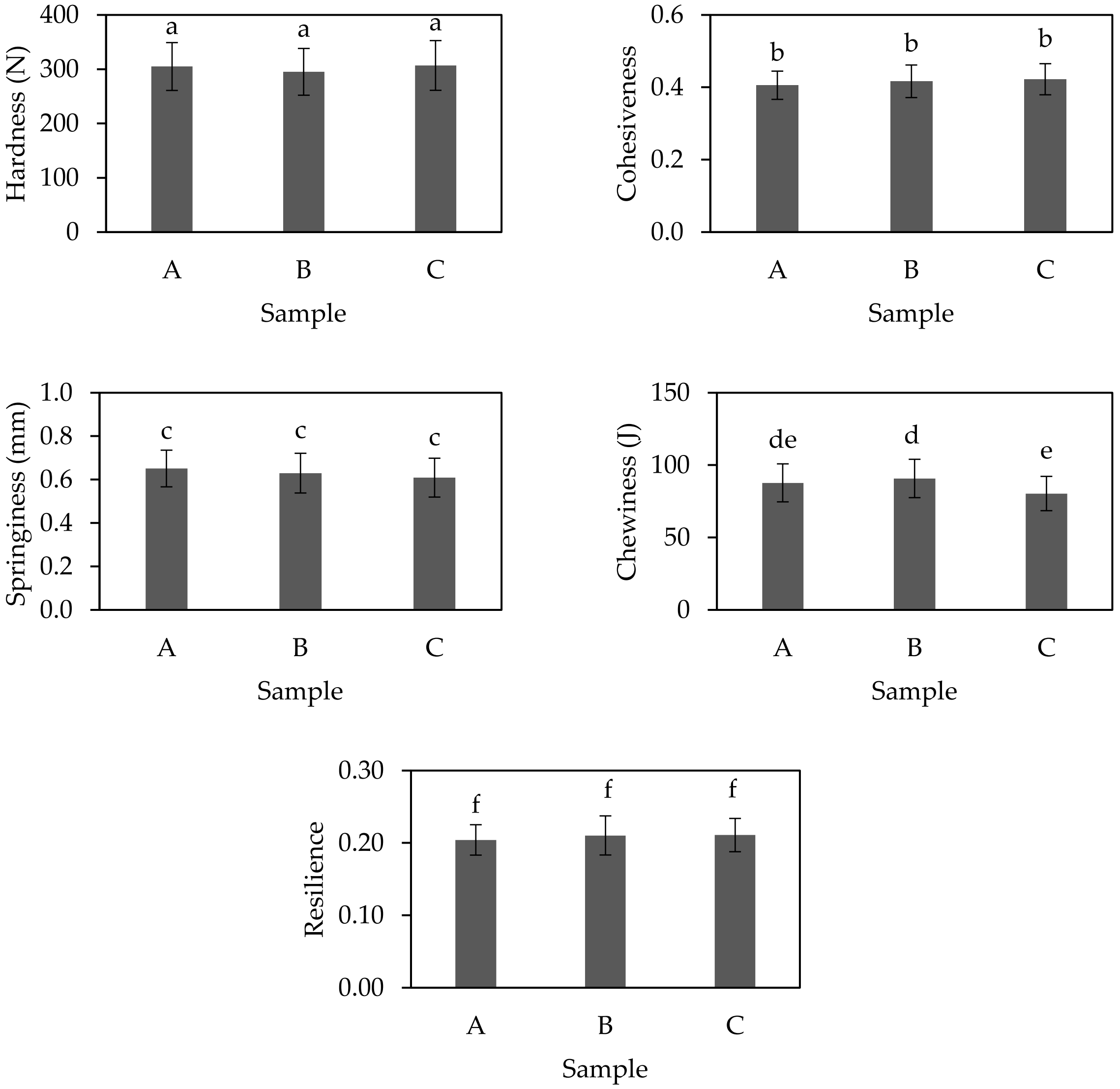

Figure S8. Participants (n=17) perception of the hardness of three black bean samples (A: PEF and thermally processed without $CaCl_2$ addition; B: PEF and thermally processed with $CaCl_2$ addition; and C: No PEF, thermally processed with $CaCl_2$) rated on a five-point hedonic scale.

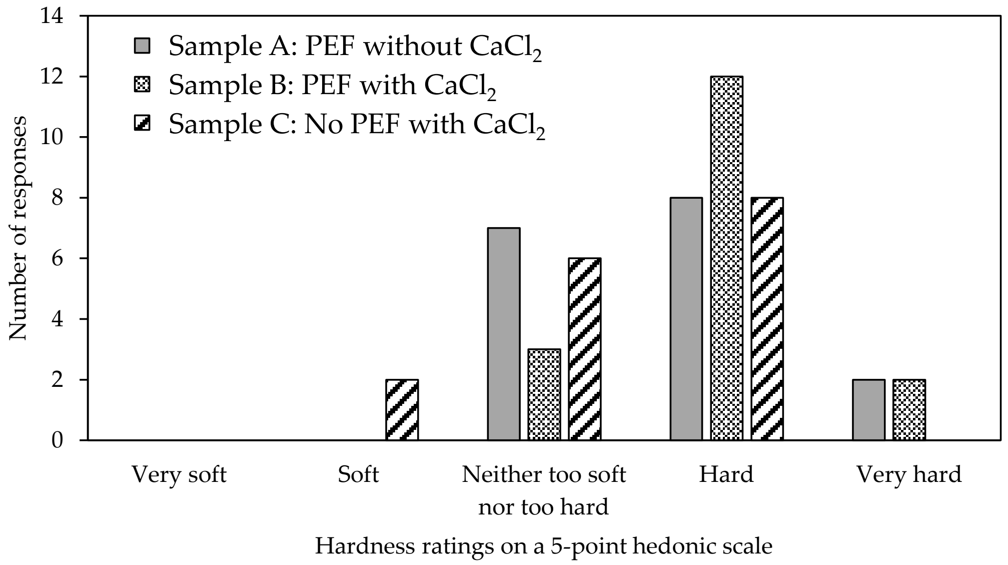

Figure S9. Boxplot of the chewing duration of the three samples (A: PEF and thermally processed without $CaCl_2$ addition; B: PEF and thermally processed with $CaCl_2$ addition; and C: No PEF, thermally processed with $CaCl_2$) by 17 participants. Upper and lower lines of the box are the upper and lower quartiles, the lines inside the box represent the median values, and the lines extending outside of the box are the minimum and maximum values. No significant difference ($p > 0.05$) was observed between samples.

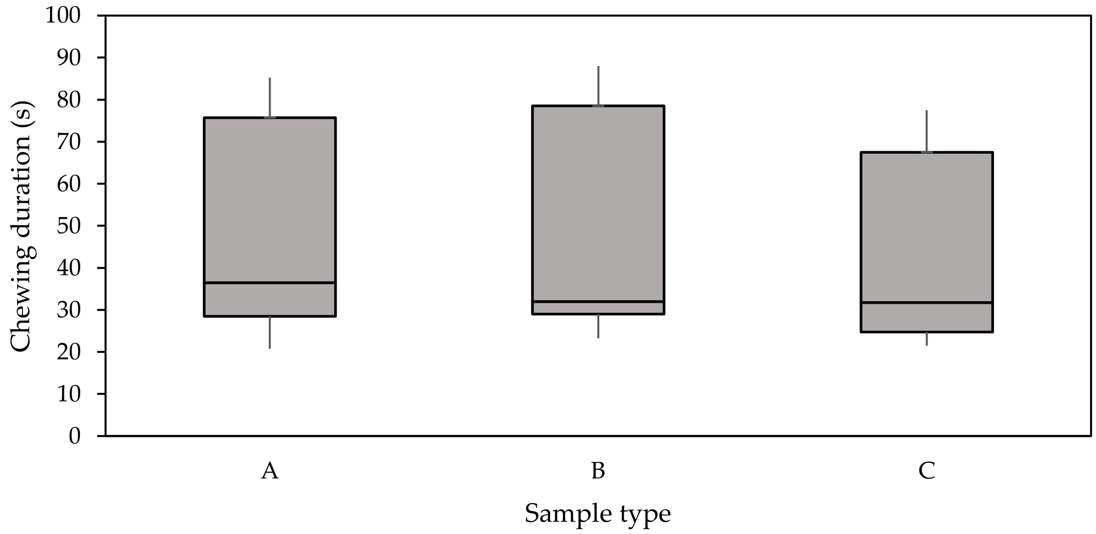

Figure S10. Histograms showing the distribution of the average Rosin-Rammler parameters (x_{50} and b) of "all" particles of the three black bean samples (A: PEF and thermally processed without $CaCl_2$ addition; B: PEF and thermally processed with $CaCl_2$ addition; and C: No PEF, thermally processed with $CaCl_2$) after in vivo mastication (n=17).

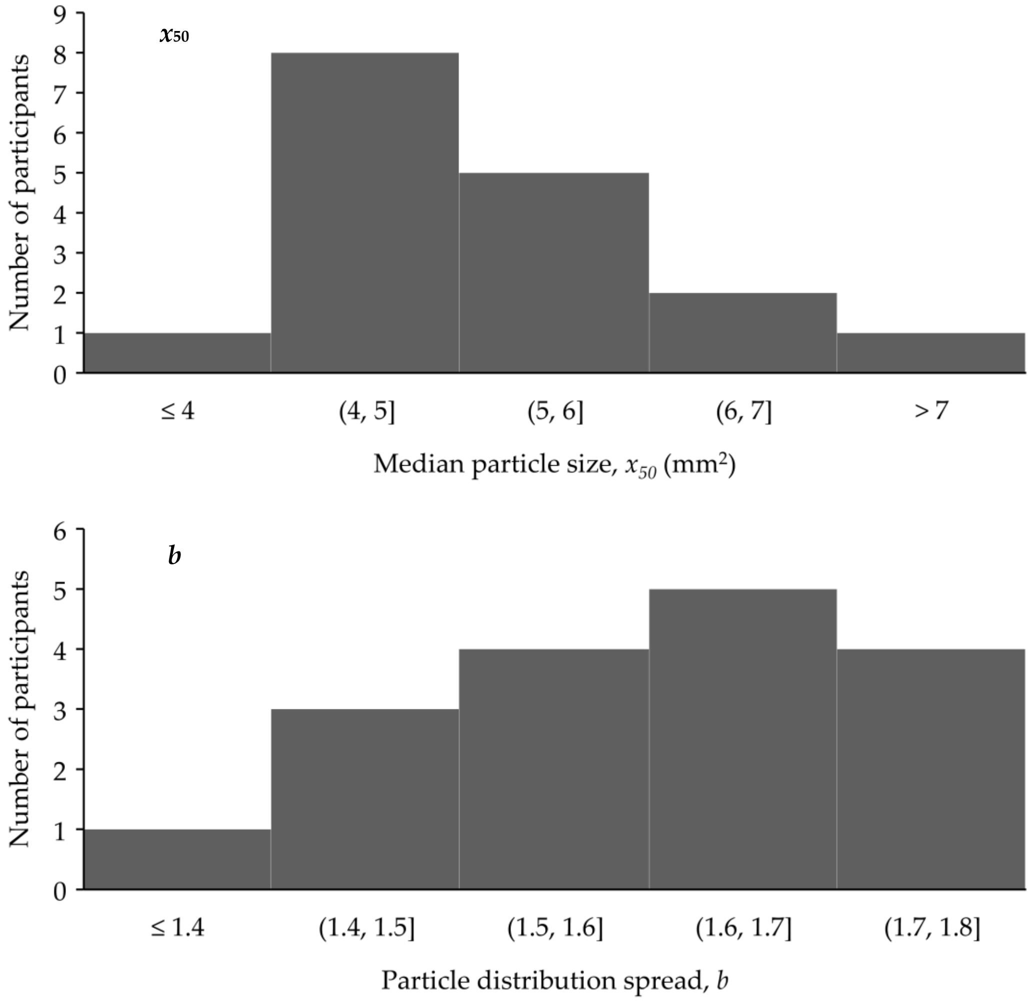

Figure S11. Light microscopic image showing broken free starch granules from the oral bolus of a slow chewer. Image were viewed under 50× magnification. Scale bar = 100 μm.

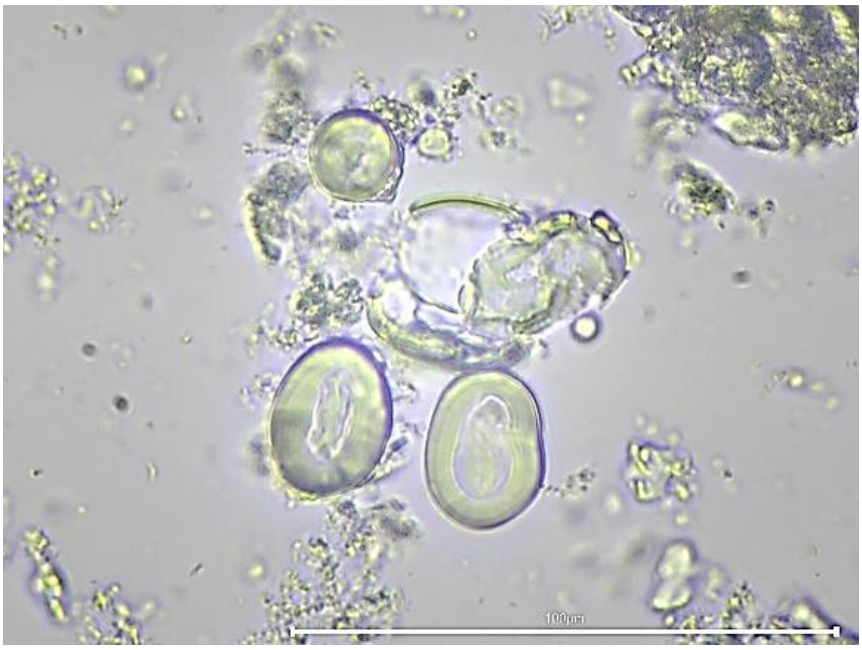

Table S1. *p*-value for the estimates of the model parameters examining the response surface effect of input variables (PEF electric field strength, PEF energy input, and calcium concentration during PEF and thermal processing) against texture parameters of cooked black beans (hardness, cohesiveness, springiness, chewiness, and resilience).

Model term corresponding to the estimated parameter	Hardness	Cohesiveness	Springiness	Chewiness	Resilience
Electric field strength (E, kV/cm)	>0.05	>0.05	>0.05	0.0002*	>0.05
Energy input (W, kJ/kg)	>0.05	>0.05	>0.05	<0.0001*	>0.05
Calcium concentration (Ca, ppm)	>0.05	>0.05	>0.05	<0.0001*	>0.05
E^2	>0.05	>0.05	>0.05	0.0017*	0.0004*
E × W	>0.05	>0.05	>0.05	<0.0001*	>0.05
W^2	>0.05	>0.05	>0.05	<0.0001*	>0.05
E × Ca	>0.05	>0.05	>0.05	>0.05	>0.05
W × Ca	>0.05	0.0384*	>0.05	0.0380*	>0.05
Ca^2	>0.05	>0.05	>0.05	>0.05	>0.05

* *p*-value <0.05 tests the null hypothesis indicating the true estimated parameter value is not zero. Changes in the input parameter is likely to be associated with changes in the texture parameter.
